# 2D- and 3D-QSAR studies of a series of benzopyranes and benzopyrano[3,4b][1,4]-oxazines as inhibitors of the multidrug transporter P-glycoprotein

**DOI:** 10.1007/s10822-013-9635-9

**Published:** 2013-02-12

**Authors:** Ishrat Jabeen, Penpun Wetwitayaklung, Peter Chiba, Manuel Pastor, Gerhard F. Ecker

**Affiliations:** 1 Department of Medicinal Chemistry, University of Vienna, Althanstrasse 14, 1090 Vienna, Austria; 2 Institute of Medical Chemistry, Medical University of Vienna, Waehringerstrasse 10, 1090 Vienna, Austria; 3 Research Unit on Biomedical Informatics (GRIB), IMIM/Universitat Pompeu Fabra, Dr. Aiguader 88, 08003 Barcelona, Spain

**Keywords:** P-glycoprotein, Multidrug resistance, QSAR, GRIND

## Abstract

The ATP-binding cassette efflux transporter P-glycoprotein (P-gp) is notorious for contributing to multidrug resistance in antitumor therapy. Due to its expression in many blood-organ barriers, it also influences the pharmacokinetics of drugs and drug candidates and is involved in drug/drug- and drug/nutrient interactions. However, due to lack of structural information the molecular basis of ligand/transporter interaction still needs to be elucidated. Towards this goal, a series of Benzopyranes and Benzopyrano[3,4b][1,4]oxazines have been synthesized and pharmacologically tested for their ability to inhibit P-gp mediated daunomycin efflux. Both quantitative structure–activity relationship (QSAR) models using simple physicochemical and novel GRID-independent molecular descriptors (GRIND) were established to shed light on the structural requirements for high P-gp inhibitory activity. The results from 2D-QSAR showed a linear correlation of vdW surface area (Å^2^) of hydrophobic atoms with the pharmacological activity. GRIND (3D-QSAR) studies allowed to identify important mutual distances between pharmacophoric features, which include one H-bond donor, two H-bond acceptors and two hydrophobic groups as well as their distances from different steric hot spots of the molecules. Activity of the compounds particularly increases with increase of the distance of an H-bond donor or a hydrophobic feature from a particular steric hot spot of the benzopyrane analogs.

**Electronic supplementary material:**

The online version of this article (doi:10.1007/s10822-013-9635-9) contains supplementary material, which is available to authorized users.

## Introduction

Development of multidrug resistance (MDR) is one of the major challenges in cancer chemotherapy, as it limits the effectiveness of many clinically important agents [1]. One of the basic underlying mechanisms is overexpression of the mdr1 gene product, P-glycoprotein (P-gp) [2], which belongs to the ATP-binding cassette (ABC) family of transporters [3]. It is highly promiscuous in its ligand recognition profile and thus transports a large variety of structurally and functionally diverse compounds out of tumor cells [4]. Apart from its role in tumor cells it is expressed at epithelial cells of liver, kidney, intestine and colon, as well as at the blood brain barrier. Thus, P-gp not only plays an important role in maintaining a concentration gradient of toxic compounds at these physiological barriers, but also modulates the pharmacokinetics of drugs that are recognized as P-gp substrates.

Within the past decade numerous inhibitors of P-gp mediated drug efflux have been identified [3]. Several compounds entered even phase III clinical studies, such as MS-209 (dofequidar fumarate), tariquidar, valspodar and elacridar [5, 6]. However, none made it to the market so far, mainly because of lack of efficacy or severe side effects. In light of our extensive SAR and QSAR studies of propafenones [7, 8], benzophenones [9] and dihydrobenzopyrans [10], a new class of conformationally restricted benzopyrano[3,4b][1,4]oxazines have been synthesized and biologically tested with respect to their ability to block P-gp mediated daunomycin efflux. These new P-gp inhibitors offer the advantage of a remarkably reduced conformational flexibility, which renders them versatile molecular tools for probing stereoselective differences of drug/P-gp interaction [11], as well as for 3D-QSAR studies. These might be performed by utilizing alignment-dependent approaches, such as CoMFA and CoMSIA, or by alignment independent methods using descriptors derived from Molecular Interaction Fields (MIFs), like the GRIND [12]. In particular, the latter allow the analysis of structurally diverse data series. GRID MIFs [13] have been applied to many areas of computational drug discovery, including 3D-QSAR [14], docking [15], high-throughput virtual screening [16], ADME profiling, kinetic [17, 18] and metabolism prediction [19] of early drug candidates. In this manuscript we explore the capability of the GRIND approach to derive predictive 3D-QSAR models for a set of diastereomeric benzopyrano[3,4b][1,4]oxazines. The GRIND based 3D-QSAR models added value in recognition of important pharmacophoric features and their mutual distances. In addition, molecular shape of the P-gp inhibitors has been recognized as an important structural prerequisite for high pharmacological activity.

## Materials and methods

### Chemistry

Synthesis of the benzopyrane common scaffold was achieved in analogy to the procedure reported by Godfrey et al. [20] and following our strategy outlined recently [11]. Besides a set of diastereomeric esters and benzopyrano[3,4b][1,4]oxazines, also a set of corresponding ethers were synthesized. Respective procedures and experimental details are provided in the supplementary material. In general, compounds showing 4aS,10bR stereochemistry are denoted as (a)-series, whereas the respective 4aR,10bS-analogues are assigned as (b)-series (Table 1).

**Table 1:** Enantiomerically pure benzopyrano [3,4b][1,4] oxazines (**5a–22b**) and their IC_50_ values


#	Scaffold	Stereochemistry	R1	R2	IC_50_ μM ± SD	LogP(o/w)
**5a**	(A)	(3S,4R)	(L) CH_3_	H	29.85 ± 0.01	2.84
**5b**	(A)	(3R,4S)	(L) CH_3_	H	14.55 ± 0.05	2.84
**6a**	(A)	(3S,4R)	(L) CH(CH_3_)_2_	H	2.40 ± 0.03	3.82
**6b**	(A)	(3R,4S)	(L) CH(CH_3_)_2_	H	2.70 ± 0.02	3.82
**7a**	(A)	(3S,4R)	(L) CH_2_(C_6_H_5_)	H	0.55 ± 0.02	4.38
**7b**	(A)	(3R,4S)	(L) CH_2_(C_6_H_5_)	H	0.77 ± 0.04	4.38
**8a**	(A)	(3S,4R)	(L) CH_3_	CH_3_	3.96 ± 0.06	3.11
**8b**	(A)	(3R,4S)	(L) CH_3_	CH_3_	3.72 ± 0.03	3.11
**9a**	(A)	(3S,4R)	(L) CH(CH_3_)_2_	CH_3_	0.96 ± 0.06	4.08
**9b**	(A)	(3R,4S)	(L) CH(CH_3_)_2_	CH_3_	1.35 ± 0.003	4.08
**10a**	(A)	(3S,4R)	(D) CH(CH_3_)_2_	H	4.62 ± 0.31	3.81
**10b**	(A)	(3R,4S)	(D) CH(CH_3_)_2_	H	1.34 ± 0.08	3.81
**11a**	(A)	(3S,4R)	(D) CH(CH_3_)_2_	CH_3_	1.01 ± 0.02	4.08
**11b**	(A)	(3R,4S)	(D) CH(CH_3_)_2_	CH_3_	1.00 ± 0.05	4.08
**12a**	(B)	(2S,4aS,10bR)	CH_3_	H	1241.6 ± 0.04	1.98
**12b**	(B)	(2S,4aR,10bS)	CH_3_	H	76.89 ± 0.06	1.98
**13a**	(B)	(2S,4aS,10bR)	CH(CH_3_)_2_	H	15.32 ± 0.32	2.94
**13b**	(B)	(2S,4aR,10bS)	CH(CH_3_)_2_	H	59.33 ± 0.60	2.94
**14a**	(B)	(2S,4aS,10bR)	CH_2_(C_6_H_5_)	H	2.68 ± 0.18	3.51
**14b**	(B)	(2S,4aR,10bS)	CH_2_(C_6_H_5_)	H	259.78 ± 0.06	3.51
**15a**	(B)	(2S,4aS,10bR)	CH_3_	CH_3_	47.83 ± 0.91	2.24
**15b**	(B)	(2S,4aR,10bS)	CH_3_	CH_3_	28.93 ± 0.15	2.24
**16a**	(B)	(2S,4aS,10bR)	CH(CH_3_)_2_	CH_3_	47.51 ± 0.40	3.21
**16b**	(B)	(2S,4aR,10bS)	CH(CH_3_)_2_	CH_3_	16.70 ± 0.20	3.21
**17b**	(B)	(2R,4aR,10bS)	CH(CH_3_)_2_	H	9.63 ± 0.08	2.95
**18a**	(B)	(2R,4aS,10bR)	CH(CH_3_)_2_	CH_3_	79.27 ± 0.05	3.22
**18b**	(B)	(2R,4aR,10bS)	CH(CH_3_)_2_	CH_3_	27.84 ± 0.02	3.22
**19a**	(C)	(2S,3S,4R)	–	H	54.05 ± 0.31	2.14
**19b**	(C)	(2S,3R,4S)	–	H	102.64 ± 0.15	2.14
**20a**	(C)	(2S,3S,4R)	–	CH_3_	5.46 ± 0.24	2.14
**20b**	(C)	(2S,3R,4S)	–	CH_3_	6.84 ± 0.15	2.14
**21a**	(D)	(2S,4aS,10bR)	–	H	48.80 ± 0.09	3.10
**21b**	(D)	(2S,4aR,10bS)	–	H	44.00 ± 0.03	3.10
**22a**	(E)	(2S,3S,4R)	–	CH_3_	35.22 ± 0.02	3.66
**22b**	(E)	(2S,3R,4S)	–	CH_3_	45.17 ± 0.05	3.66

## Calculation of physicochemical parameters

### Hansch analysis

Molecular descriptors supplied by the program MOE (atom and bond counts, connectivity indices, partial charge descriptors, pharmacophore feature descriptors, logP (o/w), calculated physical property descriptors) were computed for Hansch analysis. QSAR-Contingency [21], a statistical application in MOE, was used for the selection of relevant descriptors. PLS analysis was performed to determine the relationship between these 2D molecular descriptors and biological activity of the compounds. The predictive ability of the model was determined by classical leave one out (LOO) and leave one pair out cross validation procedures (SM Table 1). In order to remove any bias, the final model was externally validated by using a test set of already published dihydrobenzopyrans [10].

### GRIND

3D conformations of the molecules in the data set were obtained from their 2D coordinates by using the program CORINA [22]. Molecular Interaction Fields (MIF) were calculated as GRID based fields in Molecular Discovery software Pentacle [23] using four different probes: DRY probe to represent hydrophobic interactions, O sp² carbonyl oxygen probe to represent H-bond donor feature of the molecules, N1 probe to represent –NH which is a neutral flat probe as an H-bond acceptor in the molecules and the TIP probe that represents the shape of the molecule, in terms of steric hot spots. The regions with the most relevant MIF were extracted by applying the AMANDA algorithm [24] that uses the intensity of the field at a node and the mutual node–node distances between the chosen nodes. At each point, the interaction energy (*Exyz*) was calculated as a sum of Lennard-Jones energy (Elj), Hydrogen bond (Ehb) and Electrostatic (Eal) interactions.

$$ Exyz = \sum {E{\text{lj}}} + \sum {E{\text{el}}} + \sum {E{\text{hb}}} $$

Default values of probe cutoff (DRY = −0.5, O = −2.6, N1 = −4.2, TIP = −0.74) was used for discretization of MIF. Nodes with an energy value below this cutoff were discarded. The Consistently Large Auto and Cross Correlation (CLACC) algorithm [23] was used for encoding the prefiltered nodes into GRIND thus producing most consistent variables as compared to MACC [25]. The values obtained from this analysis were represented directly in correlogram plots, where the product of node–node energies is reported versus the distance separating the nodes. Highest energy product can be defined for the same probe (obtaining four auto correlograms: DRY–DRY, O–O, N1–N1 and TIP–TIP) and for pairs of different probes (obtaining six cross correlograms: DRY–O, DRY–N1, DRY–TIP, O–N1, O–TIP, and N1–TIP). The QSAR model was built using PLS and its quality assessed by means of *q*² and standard deviation error of prediction (SDEP). Classical leave one out (LOO) method was applied to calculate *q*² values. The final model was validated by leave one pair out cross validation as already described in the 2D-QSAR section (SM Table 2) as well as by an external test set composed of previously published compounds.

### Pharmacology

Biological activity of target compounds **5a–22b** was assessed using the daunorubicin efflux protocol as described previously [26]. Briefly, multidrug resistant CCRF-CEM vcr 1,000 cells were incubated with daunorubicin and the decrease in mean cellular fluorescence in dependence of time was measured in presence of various concentrations of the modulator. IC_50_ values were calculated from the concentration–response curve of efflux *V*_*max*_ */K*_*m*_ versus concentration of the modulator. Thus, the effect of different modulators on the transport rate is measured in a direct functional assay. Values are given in Table 1 and are the mean of at least three independently performed experiments. Generally, inter experimental variation was below 20 %.

## Results and discussion

### Structure activity relationships (SAR)

Biological activity values of the data series cover a range of more than three orders of magnitude (Table 1) with the two phenylalanine esters **7a** and **7b** being the most active compounds (**7a**: 0.55 μM; **7b**: 0.77 μM), followed by *N*-methylated l-valine analogues **9a** (0.96 μM) and **9b** (1.35 μM), which are by a factor of 2 more active than the corresponding unsubstituted analogs **6a** (2.40 μM) and **6b** (2.70 μM). The same trend could be observed for the respective d-valine derivatives. This observation is even more pronounced for the alanine derivatives (compare methylated analogs **8a** (3.96 μM) and **8b** (3.72 μM) versus respective secondary amines **5a** (29.85 μM) and **5b** (14.55 μM). This most probably is due to a logP effect with more lipophilic compounds showing higher biological activity, which has been shown for numerous classes of P-gp inhibitors [27].

It has to be noted that for all seven diastereoisomeric pairs showing a bicyclic scaffold almost no differences in biological activity exist. However, this pattern changes remarkably upon ring closure to the tricyclic benzopyrano[3,4b][1,4]oxazines. While all stereoisomers containing a valine moiety (**13a,b**; **16a,b**, **18a,b**, **19a,b**–**22a,b**) are still within one order of magnitude, both the alanine and phenylalanine derivatives exhibit remarkable differences in their P-gp inhibitory potency (IC_50_) values. Interestingly, in case of alanine, the 4aS,10bR-isomer **12a** is by a factor of 15 less active than the diastereomeric 4aR,10bS analogue **12b**, whereas in case of the phenylalanine derivatives this behavior reverses with the 4aS,10bR-isomer **14a** being by two orders of magnitude more active than **14b**. This difference in their biological activities might be due to difference in mode of interaction of diastereoisomeric pairs as has been indicated in a preceding publication [11].

### Hansch analysis

3D structures of all diastereoisomers were built with the builder function of MOE 2011. 10 and energy minimised using the MMFF94 force field which uses a bond charge increment method to set the electrostatic partial charges [28]. In order to determine the influence of physicochemical properties of the compounds on their biological activity, QSAR analyses were performed by using the software package MOE version 2011. 10 and MOE’s contingency analysis tool for identification of the most important descriptors. The multiple linear regression analysis produced an equation solely based on the hydrophobic van der Waals surface area (vsa_hyd) (Eq. 1). Interestingly, descriptors related to electrostatic properties, such as topological polar surface area and molar refractivity, did not show significant contributions to the model.

1 $$ {\text{Log}}\,(1/{\text{IC}}_{50} ) = 0.01\,({\text{vsa}}\_{\text{hyd}}) - 4.74 $$

$$ {\text{n}} = 35,\,{\text{R}}^{2} = 0.67,\,{\text{q}}_{{({\text{LOO}})}}^{2} = 0.63,\,{\text{RMSE = 0}} . 4 8 $$

**Fig. 1:**
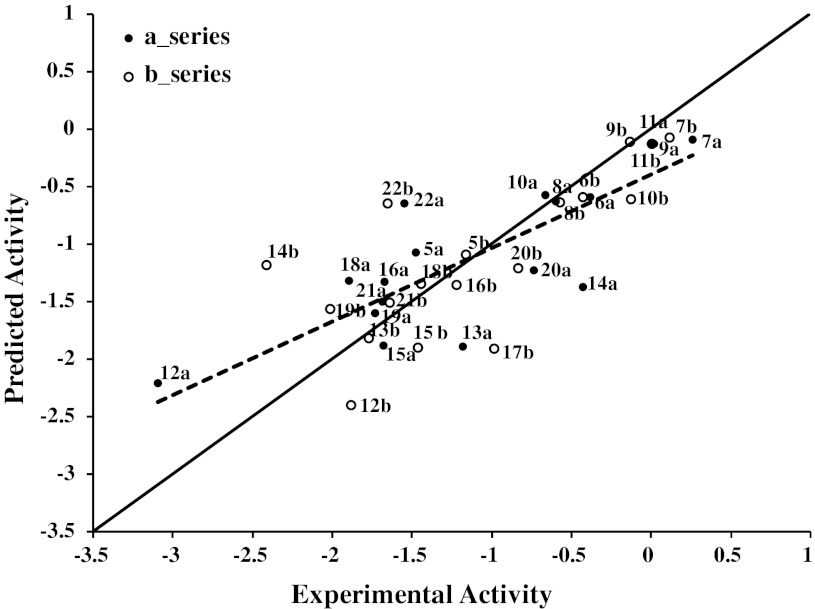
Plot of observed versus predicted MDR-modulating activity expressed as log 1/IC_50_ (*dashed line*). Predicted values were obtained with leave-one-out cross validation procedure. Results of linear regression between observed and predicted log 1/IC_50_ values (*dashed line*) and 1:1 line (*continuous line*) are shown on the plot for comparative evaluation

Figure 1 shows a plot of observed versus biological activity predicted by QSAR E. 1. Compounds **14b** and **22b** show outlier behaviour (residual value above one log unit). Upon removal of these two compounds, the *q*² value improves to 0.70. Interestingly, both compounds belong to the (b) series of diastereoisomers, suggesting that for this series additional factors other than lipophilicity might play a role. Vsa_hyd describes the sum of van der Waals surface areas of hydrophobic atoms (Å^2^). This is perfectly in line with previous studies which showed that distribution of hydrophobicity within the molecules influences their mode of interaction with P-gp [29] and lipophilicity needs to be considered as a space directed property [30, 31]. This space-directedness might be indicative for different orientations of molecules within the binding area of P-gp, which is mainly hydrophobic [32]. The QSAR model was further validated by using an external test set of already published dihydrobenzopyrans and tetrahydroquinolines [10]. All compounds are predicted well, with the residuals being less than one log unit from their experimental inhibitory potencies (log IC_50_). This further strengthens the reliability of the final QSAR model (Table 2). Additionally, 18 different models were developed by taking one pair of diastereoisomer out at each step. All models showed *q* ^2^ values in the range of 0.57–0.70, which further demonstrates the consistency of the QSAR model (SM Table 1).

**Table 2:** Test set of dihydrobenzopyrans, their experimental and predicted inhibitory potencies (log1/IC_50_) by 2D-QSAR and GRIND models

#	R1	Exp. log 1/IC_50_	Pred. log 1/IC_50_ (2D-QSAR)	Pred. log 1/IC_50_ (GRIND)	Log P(o/w)
1a		−1.77	−1.38	−0.79	3.19
1b		0.19	−0.22	−0.11	2.98
1c		−3.16	−2.35	−2.06	1.40
1d		−1.67	−1.28	−0.77	2.38
1e		−0.38	0.17	−0.27	4.43
2a		−0.51	−0.34	−0.02	4.27
2b		0.37	0.80	0.28	4.07
2f		−0.90	−0.57	−0.72	3.59
2g		−0.29	1.07	−0.25	4.70
3f		−0.75	−0.57	−0.77	4.34
3g		0.47	1.07	0.01	5.44

### GRID Independent molecular descriptor (GRIND) analysis

**Fig. 2:**
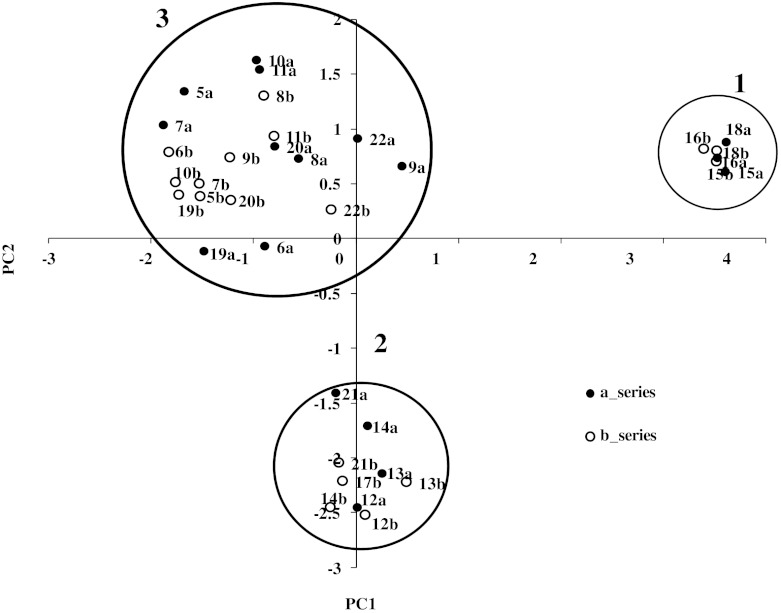
PCA score plot shows the whole series organized in three different types of inhibitors of P-gp, overall no outlier has been observed in the dataset

The previously computed molecular structures along with their activity values (expressed as log1/IC_50_) were loaded into the software package Pentacle (v 1.06) [23] to derive 3D-QSAR model using GRIND descriptors. According to previous findings for propafenone analogs, all compounds were modeled in their neutral form [33]. Structural variance of the data was analyzed with principal component analysis (PCA) performed on the complete set of GRIND descriptors. The first two principal components explain about 32 % of the descriptor variance in the data set. Principal component analysis (PCA) on the data matrix showed that the series is organized in three different clusters (Fig. 2). Molecules in the cluster on the right hand side (cluster 1) do not contain any H-bond donor, while the second cluster (cluster 2) contains one H-bond donor group. The 3rd cluster (cluster 3), located on the upper left corner of the plot, contains compounds with two H-bond donor groups in their structures. Furthermore, rigid and smaller compounds (cluster 1 and cluster 2) are separated from the flexible ones (cluster 3). Overall, compounds in cluster 3 are more potent than compounds in cluster 1 and 2, suggesting that an elongated structure is an important perquisite for high P-gp inhibitory potency.

In order to identify the more important pharmacophoric features of ligand–protein interaction, a PLS model was built, using the complete set of active variables (450) generated by Pentacle (v 1.06). This resulted in a one-latent variable (LV1) model with an *r* ^2^ of 0.51 and a cross-validated (LOO) *q* ^2^ value of 0.27, which was quite unsatisfactory. Thus, variable selection was applied to reduce the variable number using the FFD variables selection algorithm [34] implemented in Pentacle. This resulted in a decrease from 450 to 196 variables and a large increase of the model quality (*r*² of 0.72, *q* ^2^ of 0.58, standard error of prediction 0.52).

**Fig. 3:**
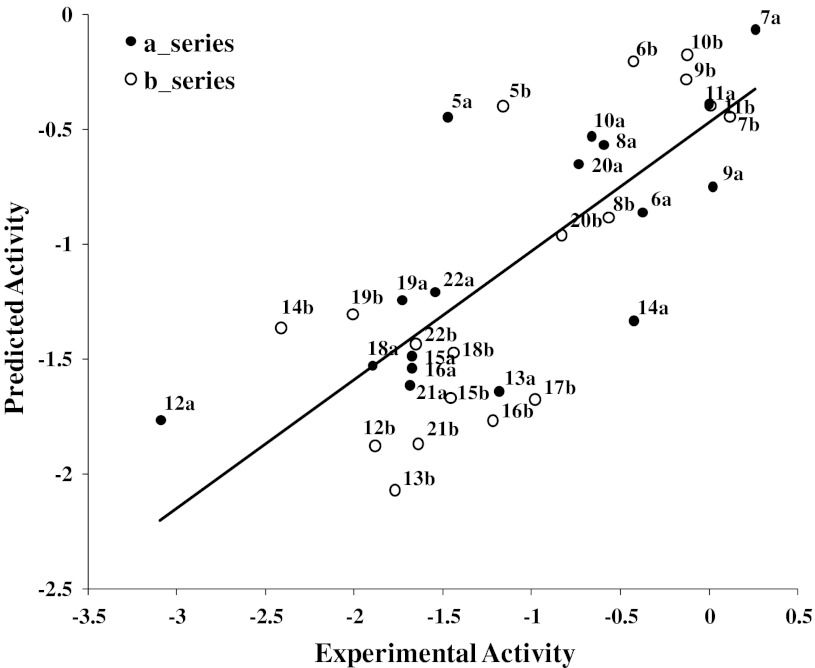
Plot of observed versus predicted (LOO) MDR-modulating activity (log1/IC_50_) of inhibitors of P-gp obtained with the GRIND descriptors

**Fig. 4:**
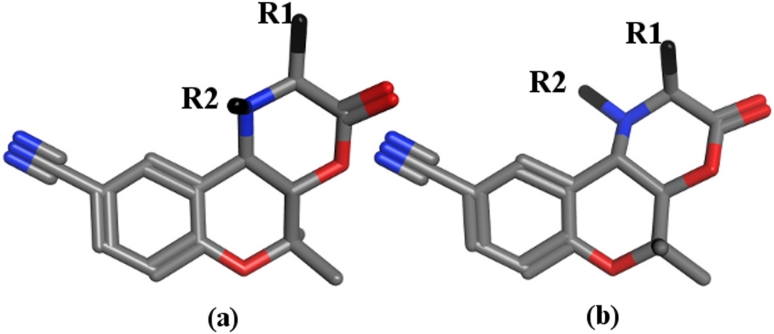
3D representatives of *series (a)* having 2S,4aS,10bR-configuration and *series (b)* having 2S,4aR,10bS-configuration

With the exception of compounds **14b,** **5a,** and **12a**, all compounds are within one order of magnitude from their predicted values (**14b**: obs 259.78, pred 23.21; **5a**: obs 29.85, pred 2.80; **12a**: obs 1241.65, pred 58.30 μM) (Fig. 3). The outlier behavior of these three compounds might be due to potential different interaction behavior of the two diastereomeric series as reported by Jabeen et al. [11]. However, building two separate QSAR models composed of compounds of series (a) and series (b) in two separate training sets showed an analogous picture and did not improve the results (data not shown). Thus, although GRIND descriptors are able to capture different configurations, they were not able to extract the differences of the two diastereomeric series. This might be due to the fact that the molecules are quite compact (Fig. 4) and GRIND is considering MIFs within a grid step of 0.5 Å.

All compounds of the external test set are predicted within one log unit from the experimental inhibitory potencies (log IC_50_), except (1c), where the residual is slightly more than one log unit (Table 2). The low activity of 1c mainly might be due to its low logP value, which is not properly reflected in the GRIND based pharmacophoric features. Thus, GRIND is over predicting the compound. The overall good predictive ability and model statistics of all 18 leave one pair out GRIND models further demonstrates the consistency and validity of the GRIND based 3D-QSAR model (SM Table 2).

**Fig. 5:**
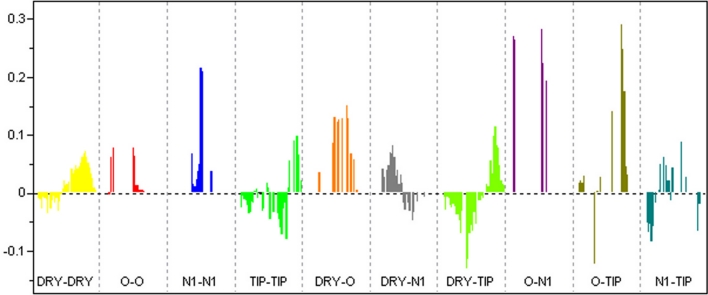
PLS Coefficients showing the descriptors directly (positive value) or inversely (negative values) correlated to IC_50_. P-gp inhibitory potency particularly increases with the increase in (N1–N1), (O–N1) and (O–TIP) descriptor value

Analysis of the PLS coefficients profile of the GRIND model allows to identify those descriptors which exhibit the largest contribution to the model. According to the bar plot shown in Fig. 5, certain distances of the N1–N1, O–N1, and O–TIP probes are participating most in explaining the variance in the biological activity values (Table 3).

**Table 3:** Summary of GRIND variables and their corresponding distances that are identified as being highly correlated to biological activity of compounds **5a–22b**

Correlogram	Distance	Comment
DRY–DRY	13.2–13.6 Å	Optimal distance separating two hydrophobic groups. More pronounced in phenylalanine derivatives
N1–N1	8.8–9.2 Å	Related to two hydrogen bond acceptor atoms in the molecules. This is mainly associated to the carbonyl group and the hydroxyl groups in tert-butyl esters
O–N1	2.4–2.8 Å	Well pronounced in tert-butyl esters with IC_50_ ~1 μM. Positive contribution towards P-gp inhibitory potency
O–N1	9.6–10.0 Å	Complements N1–N1, contributing directly to the inhibition of P-gp mediated drug efflux
O–TIP	12.8–13.2 Å	H-bond donor present far away from a steric hot spot, positive contribution to IC_50_
O–TIP	5.6–6.0 Å	H-bond donor present quite near to a steric hot spot, contributing negatively
DRY–TIP	15.2–15.6 Å	Complements to DRY–DRY correlogram, positive contribution to P-gp inhibitory potency

The sum of the van der Waals surface areas of hydrophobic atoms (vsa_hyd) has emerged as an important determinant for high biological activity of benzopyrane-type P-gp inhibitors (Eq. 1). The 3D-QSAR model using GRIND descriptors further refines this general property and identified two hydrophobic regions (DRY–DRY) separated by a certain distance range in all active compounds. These represent the aromatic ring of the benzopyrane ring system and R1. In the most active phenylalanine derivatives (**7a,b** and **14a,b**) the two regions are separated by a distance of 13.2–13.6 Å, which is considered optimal according to the GRIND model. Thus, adding a large hydrophobic group (large vsa_hyd) at the position of R1 might lead to a further increase of the biological activity.

**Fig. 6:**
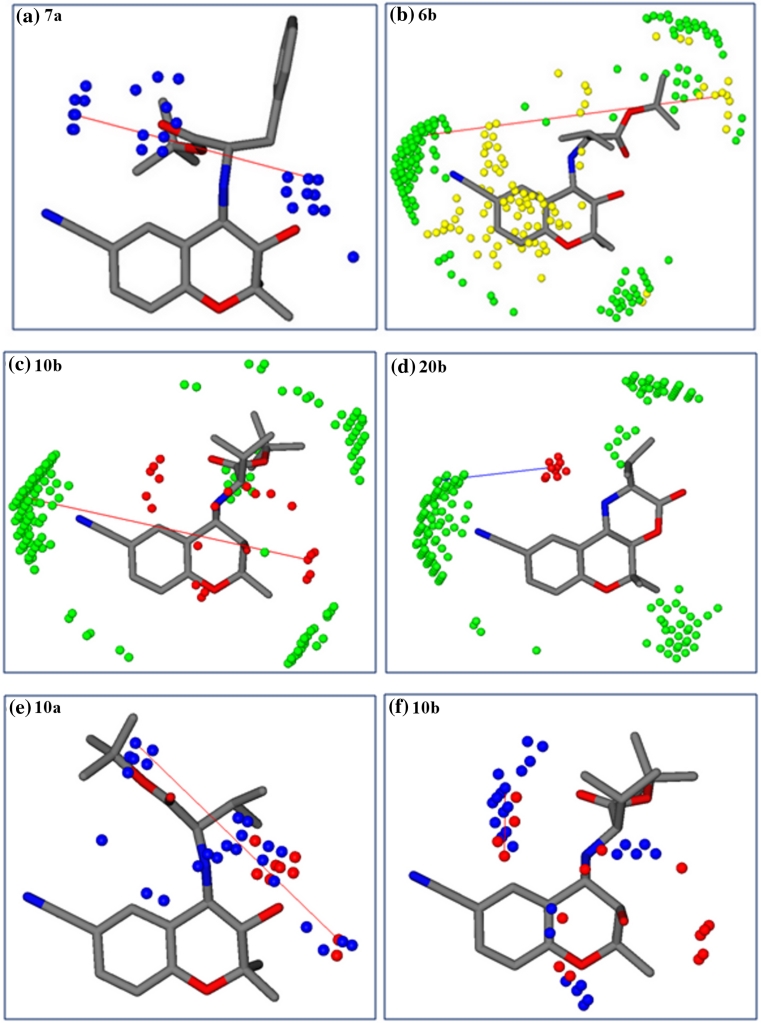
**a** Represents two H-bond acceptors (N1–N1: *blue hot spots*) at a distance of 8.8–9.2 Å. **b** DRY-TIP represents a hydrophobic probe (DRY: *yellow hot spots*) at a distance of 15.2–15.6 Å from a steric hot spot (TIP: *green region*). **c** O–TIP outline an H-bond donor (OH) (O: *red hot spots*) at a distance of 12.8–13.2 Å from the 9-carbonitril edge” of the molecule. **d** Marks an H-bond donor (–NH) at a distance of 5.6–6.0 Å from the 9-carbonitril edge of the molecule (O–TIP). **e** Representing an H-bond donor (OH) at a distance of 9.6–10.0 Å from an H-bond acceptor (C=O), present only in esters (O–N1). **f** Representing, H-bond donor (–NH) at a distance of 2.4–2.8 Å from an H-bond acceptor (C=O)

Previous QSAR studies on propafenone derivatives have demonstrated the importance of H-bond acceptors and their distance from the central aromatic ring [35, 36]. Furthermore, Seelig [37, 38] more explicitly defined two patterns of H-bond acceptor groups and their fixed spatial distance observed in ligands of P-gp. Pattern I contains two H-bond acceptors separated by a distance of 2.51 ± 0.30 Å, while pattern II comprises two or three H-bond acceptor groups at a distance of 4.60 ± 0.60 Å apart. Interestingly, the 3D-QSAR model based on benzopyrano[3,4b][1,4]oxazines identified an optimal distance of 8.8–9.2 Å between two H-bond acceptor groups (N1–N1) in all compounds exhibiting IC_50_ ~1 μM. The N1–N1 correlogram is mainly associated to the carbonyl group and the hydroxy group in tert-butyl esters **7a–11b**. For tricyclic compounds (**15a–16b** and **18a,b**) it is associated to the distance of the carbonyl group and the tertiary nitrogen atom. Finally, for amino alcohols **19a–20b** this descriptor refers to the two hydroxy groups in the molecules. This indicates that the presence of two H-bond acceptors is important for the biological activity of P-gp inhibitors if they are separated by a distance of ~8.8–9.2 Å (Fig. 6a), which is in line with several other studies. Crivori et al., used GRIND descriptors to identify 3D pharmacophoric features which differentiate P-gp inhibitors from substrates. They reported two H-bond acceptors at a distance of 8.0 Å apart from each other in P-gp inhibitors [39], whereas a distance of 11.5 Å between two H-bond acceptors, along with the importance of shape descriptors, have been reported by Cianchetta et al. [40] for substrates of P-gp.

However, despite of similarities in the number of H-bond acceptors necessary for a high biological activity, a direct comparison of distance matrices thereof across different chemical scaffolds reveals some differences. This most probably is due to the fact that the large binding site of P-gp has multiple spots able to contribute to H-bond interactions and that different chemical series utilize different H-bond interaction patterns.

Apart from a certain number of H-bond acceptors, one H-bond donor along with hydrophobic regions distribution have been identified as important pharmacophoric features of P-gp inhibitors/substrates [8, 36]. It is worth noting that a very similar MIF based pharmacophore of P-gp inhibitors was recently published by Broccatelli et al. [41]. They identified one H-bond acceptor and two large hydrophobic regions, together with an optimal molecular shape, as being important for high activity, and successfully used their model for virtual screening to identify new P-gp inhibitors. The results are further in line with Boccard et al. [42] outlining an optimal shape and hydrophobicity as major physicochemical parameters responsible for the affinity of flavonoid derivatives for P-gp [43, 44].

Also in our GRIND model, shape based probes (TIP) defining steric hot spots exhibit a significant contribution. Especially the 9-carbonitrile group in the benzopyrane scaffold encodes an important molecular boundary (steric hot spot) and serves as anchor for defining optimal distance ranges to an H-bond donor (O–TIP correlogram) as well as to a hydrophobic feature (DRY–TIP correlogram). The O–TIP combination of probes encodes the shape of the molecules (steric hot spots) together with an H-bond donor group. Interestingly, O–TIP coefficients are negative for a distance between 5.6 and 6.0 Å, but become positive for larger distances (12.8–13.2 Å). These distances (12.8–13.2 Å) are present in benzopyranes bearing tert-butyl esters (**5a–11b**) and amino alcohol derivatives (**19a–20b** and **22a,b**) as shown in Fig. 6c. In tricyclic diastereoisomers (**12a–14b** and **17b**) these descriptors are linked to shorter distances and mark (–NH) as an H-bond donor at a distance of 5.6–6.0 Å apart from the cyano group, which is the main group contributing to the TIP MIF, and seems to be related with a negative influence for the biological activity of this group (Fig. 6d). This indicates that, in general, the most potent P-gp inhibitors show extended conformations and have an H-bond donor group far from regions with a strong TIP probe related field.

Analyzing the DRY-TIP correlogram it becomes evident that a hydrophobic group at a distance of 15.2–15.6 Å from one of the “edges” of the molecule (steric hot spot, cyano group) positively contributes to biological activity. In tert-butyl esters (**5a–11b**) and **14a,b** these two probes map the distance between a hydrophobic group (R_1_) (**14a,b**) or tert-butyl group in **5a–11b** from the cyano group (Fig. 6b). In analogy to the O–TIP correlogram, DRY–TIP shows a negative contribution towards biological activity for shorter distances (7.6–8.0 Å) of these probes.

Finally, the O–N1 correlogram (H-bond donor–H-bond acceptor) points towards two positive contributions at a distance of 9.6–10.0 and 2.4–2.8Å, respectively (Fig. 6e, f). The first distance is linked to the hydroxyl and carbonyl group in **5a–11b** and is complementary to the N1–N1 correlogram as already discussed. The second distance refers to the –NH and carbonyl group. O–N1 probes at both distance ranges are well pronounced in tert-butyl esters (**5a–11b**) as well as in amino alcohol substituted derivatives (**19a–20 b** and **22a,b**). However, in all tricyclic compounds (**12a–18b**) the two probes do not fit either of the distance ranges.

To summarize, the presence of two H-bond-acceptor groups and one H-bond donor at a particular distance from each other and from a particular “edge” or steric hot spot of the molecule is associated to an increase of the biological activity in benzopyrane-type P-gp inhibitors (Fig. 6).

## Conclusions

Benzopyrano-[3,4b][1,4]oxazines are versatile molecular tools for probing the stereoselectivity of P-glycoprotein. For a distinct substitution pattern, different pairs of diastereoisomers exhibit a large difference in their potency to inhibit P-gp mediated drug efflux. Unfortunately, GRIND-based 3D-QSAR models were unable to link these differences to concrete differences of distances between pharmacophoric hot spots, even if the GRIND analysis provided a reasonably well performing 3D-QSAR model outlining a set of important pharmacophoric features. Two H-bond-acceptor groups, one H-bond donor at a particular distance from each other as well as distinct distances of these probes to steric hot spots seem to play a major role in the interaction of benzopyrane-type P-gp inhibitors. The activity particularly increases when increasing the distance between an H-bond donor or a hydrophobic feature and a particular steric hot spot of the benzopyrane analogs. This not only further highlights the importance of H-bonding, but also indicates that a certain shape/configuration of the molecules is important for high activity. Further analyses will focus on a generalisation of this finding in other series of P-gp inhibitors.

## Electronic supplementary material

Below is the link to the electronic supplementary material.

Supplementary material 1 (DOCX 138 kb)

## Abbreviations

P-gp: P-glycoprotein
MDR: Multidrug resistance
ABC: ATP binding cassette
QSAR: Quantitative structure–activity relationship
GRIND: GRID-independent molecular descriptors
MIF: Molecular interaction field
