# Supplementary material for: 2D- and 3D-QSAR studies of a series of benzopyranes and benzopyrano[3,4b][1,4]-oxazines as inhibitors of the multidrug transporter P-glycoprotein
Source: J Comput Aided Mol Des. 2013 Feb 12;27(2):161–71. doi: 10.1007/s10822-013-9635-9 (PMC3589648; doi:10.1007/s10822-013-9635-9)
Supplement: Supplementary file 1 — Supplementary material 1 (DOCX 138 kb) [file 10822_2013_9635_MOESM1_ESM.docx]

Electronic Supplementary Material for Journal of Computer Aided Molecular Design

Electronic Supplementary Information

2D- and 3D-QSAR Studies of a Series of Benzopyranes and Benzopyrano[3,4b][1,4]-oxazines as Inhibitors of the Multidrug Transporter P-glycoprotein

IshratJabeen^a^, PenpunWetwitayaklung^a^, Peter Chiba^b^_,_ Manuel Pastor^c^ and Gerhard F. Ecker^a,^*

^a^University of Vienna, Department of Medicinal Chemistry, Althanstrasse 14, 1090 Vienna, Austria

^b^Medical University of Vienna, Institute of Medical Chemistry, Waehringerstrasse 10, 1090, Vienna, Austria

^c^Research Unit on Biomedical Informatics (GRIB), IMIM/Universitat Pompeu Fabra, Dr. Aiguader 88, E-08003 Barcelona, Spain

Phone: +43-1-4277-55110, fax: +43-1-4277-9551, e-mail: [Gerhard.f.ecker@univie.ac.at](mailto:Gerhard.f.ecker@univie.ac.at)

**Chemistry**

O-alkylation of 4-hydroxybenzonitril **(1)** with 3-trifluoroacetyl-3-methyl-but-1-yne followed by thermal cyclization gave 6-cyano-2,2-dimethyl-2H-1-benzopyrane **3**. Enantioselective epoxidation was performed in analogy to the method published by Lee *et al* [1]. The enantioselective epoxidation of benzopyranes with Jacobsons Mn(III) Salen catalyst and commercial household bleach (sodium hypochlorite) as a stoechiometric oxygen source yielded (S,S)- and (R,R)-epoxides **4a** and **4b,** respectively (Scheme 1). Enantiomeric purity of both epoxides was confirmed by HPLC analysis, using a LiChroCART (R,R)-Whelk-01 column (25x0,4 cm) and n-hexane/isopropanol (95:5) as eluent.

**Scheme 1.** Synthesis of the benzopyrane ring system and enantiomeric pure (S,S)- and (R,R)-epoxide **4a,b; (i)** DBU, CuCl_2_, -4 °C, Ar atmosphere; **(ii.a)** (S,S)-Mn(III)salen, NaOCl solution, buffer to pH 11.3, 0 °C; **(ii.b)** (R,R)-Mn(III)salen, NaOCl solution, buffer to pH 11.3, 0 °C.

Nucleophilic ring opening of these epoxides with L- and D-amino acid t-butyl esters is regioselective and stereoselective, thus giving optically pure *trans* 3,4-disubstituted benzopyranes. Tert-butyl esters of L-alanine, L- and D-valine as well as L-phenylalanine were reacted with each epoxide enantiomer to give the diastereomeric esters **5a -7b**, and **10a,b,** respectively. **5a-6b** and **10a,b** were further N-methylated to yield **8a-9b** (derived from L-alanine and -valine) and **11a,b (**derived from D-valine). All tert-butyl-esters (**5a-11b**) were hydrolyzed with 70% HClO_4_ [2] to yield the corresponding acids which were subsequently cyclisized without further purification using bis-(2-oxo-3-oxazolidinyl) phosphinic chloride, 4-dimethylaminopyridine and triethylamine to yield the target compounds **12a-18b** (Scheme 2). In the subsequent sections, compounds derived from epoxide enantiomers **4a** and **4b** are classified as series (a) and series (b), respectively.

Scheme 2. Synthesis of target compounds 12a-18b; (iii) 96% ethanol, reflux; (iv) Acidic menthol, paraformaldehyde, sodium cyanoborohydride (v) 70% HClO4; (vi) 4-dimethylaminopyridine, bis-(2-oxo-3-oxazolidinyl)-phosphinic chloride, triethylamine, dichloromethane.

For means of comparison, also a set of corresponding ethers were synthesized. The solution of both enantiomers of epoxides **4a,b** in 96% ethanol were reacted with L-valinol to yield amino alcohol substituted 2H-1-benzopyrane-3-ols **19a,b.** N-methylation as described before gave the tertiary amines **20a,b**. Valinol analogs**19a,b** were successfully cyclized by mesylation followed by intramolecular O-alkylation to yield **21a,b**. Surprisingly, in case of the tertiary amine (**20a,b**), the cyclization failed and the respective chloro derivatives **22a,b** were obtained (Scheme 3).

**Scheme 3.** Synthesis of target compounds **19a-22b; (vii)** 96% ethanol at 65°C reflux for 5 days; (**viii**) Trimethylamine, triethylamine hydrochloride, and solution of methane sulfonyl chloride in small amount of toluene at 0°C.

Experimental Section

Chemistry

Melting points of all compounds were determined on Kofler hot plate apparatus and are uncorrected. Infrared spectra were recorded on a Perkin Elmer 298 spectrometer, a BrukerAvanceDpx 200 spectrometer, a Varian Unity plus 300 spectrometer. ^1^H NMR spectra were referenced to tetramethylsilan internal standard (δ 0.0); ^13^C NMR spectroscopy CDCl_3_ served as the internal standard (δ 77.0) and on a BrukerAM 360 L spectrometer. An asterisk indicates peaks of double intensity. GC-MS spectra were recorded on a HP 5890A gas chromatography / HP 5970 mass detector. Optical rotations were measures on a Perkin Elmer 241 polarimeter in a standardised cuvette. Flash chromatography was carried out on MERCK silica gel 60, TLC on plastic sheets (Merck silica gel 60 F254).

**General procedure for the enantiomerically pure (S,S)- (4a) and (R,R)- epoxide (4b).** Commercial household bleach (DanKlorix^®^) was buffered to pH 11.3 with 0.05 N Na_2_HPO_4_ and 1N NaOH and then cooled to 0°C. To 1000 mL of this solution a solution of **3** (75.58 mmol) and Mn(III)salen catalyst (2.74x10^-3^mmol) in 76 mL of CH_2_Cl_2_ was added, stirred at 0°C for 5 hr and then at room temperature overnight. The mixture was filtered through Celite and the organic phase was separated, brined once, dried (Na_2_SO_4_) and brought to dryness. Purification by flash chromatography (petroleum ether-ethylacetate; 8:2) yield 76.9% of (S,S)-**4a** and 78.9% of (R,R)-**4b** as colorless crystals; mp 133-135^o^C; ^1^H NMR (200 MHz; CDCl_3_): δ 1.28 (s, 3H, CH_3_), 1.57(s, 3H, CH_3_), 3.52 (d, 1H, J = 4.52 Hz, 3-H/ 4-H), 3.89 (d, 1H, J = 4.52 Hz, 3-H/ 4-H), 6.84 (d, 1H, J = 8.53 Hz, 8-H), 7.51 (dd, 1H, J = 2.00 Hz, J = 8.41 Hz, 7-H), 7.63 (d, 1H, J = 2.01 Hz, 5-H); ^13^C NMR (200 MHz, CDCl_3_): δ 22.99 (2-CH_3_), 25.46 (2-CH_3_), 49.34 (3-C), 62.27 (4-C), 74.64 (2-C), 104.27 (6-C), 118.70 (CN), 119.00 (8-C), 121.67 (4a-C), 133.77, 134.38 (5-C, 7-C), 156.45 (8a-C); IR (KBr): 2227 (CN) cm^-1^, 1280 (epoxide) cm^-1^.

**General procedure for amino acid-tert-butyl-ester (5a–7b, 10a,b).** A solution of enantiomeric pure epoxide **4a** or **4b** (4.97 mmol) and corresponding D and L-amino acid-tert-butyl ester (5.47 mmol) in 50 mL 96% ethanol was stirred at 80^o^C for 5 days, then evaporated in vacuo. Purification by flash chromatography (petroleum ether/ethylacetate = 8/2) yield respective amino acid tert-butyl ester (**5a-11b**). Compounds **5a-7b** and **12a-14b** are already published by Jabeen*et al* [3].

**(3S,4R)-N-(6-cyano-3-hydroxy-2,2-dimethyl-2H-3,4-dihydro-1-benzopyrane-4-yl)-D-valine-tert-butyl-ester (10a).** (S,S) epoxide 4a and D-valinetert-butyl-ester gave 10a, yield (59%) as yellowish oil; [α]^20^ = (+) 182.38 (c = 0. 105, in CH_2_Cl_2_); ^1^H NMR (200 MHz, CDCl_3_): δ 0.89, 0.99 (each d, each 3H, each J = 7.03 Hz, CH(CH_3_)_2_), 1.17 (s, 3H, 2-CH_3_), 1.44 (s, 12H, 2-CH_3_, C(CH_3_)_3_), 1.86-2.05 (m, 1H, CH(CH_3_)_2_), 2.38 (br, 1H, NH), 2.76 (d, 1H, J = 4.27 Hz, N-CH-CO), 3.14 (dd, 1H, J = 9.54 Hz, 3.77 Hz, 3-H), 3.78 (d, 1H, J = 9.54 Hz, 4-H), 3.84 (d, 1H, J = 3.77 Hz, OH), 6.80 (d, 1H, J = 8.53 Hz, 8-H), 7.39 (dd, 1H, J = 8.56 Hz, 1.76 Hz, 7-H), 7.94 (d, 1H, J = 1.76 Hz, 5H); ^13^C NMR (200 MHz, CDCl_3_): δ 17.67 (CH-CH_3_), 19.06 (2-CH_3_), 19.77 (CH-CH_3_), 27.04 (2-CH_3_), 27.84 (C(CH_3_)_3_)_,_ 32.65 (CH(CH_3_)_2_), 56.54 (4-C), 60.53 (N-CH-CO), 71.01 (3-C), 79.60 (C(CH_3_)_3_), 82.33 (2-C), 103.58 (6-C), 118.19 (8-C), 119.34 (CN), 123.82 (4a-C), 132.42, 133.40 (5-C, 7-C), 157.56 (8a-C), 177.98 (C=O); MS m/z 375 (0.10%, M+), 273 (36.9%), 160 (22.7%); Anal. Calcd for C_21_H_30_N_2_O_4_ %: C 67.35, H 8.07, N 7.48; found: C 67.62, H 8.27, N 7.21.

**(3R,4S)-N-(6-cyano-3-hydroxy-2,2-dimethyl-2H-3,4-dihydro-1-benzopyran-4-yl)-D-valine-tert-butyl-ester (10b).** (R,R) epoxide 4b and D-valinetert-butyl-ester gave 10b, yield (57%) as yellowish oil; [α]^20^ = (+) 27.43 (c = 0. 113, in CH_2_Cl_2_); ^1^H NMR (200M Hz, CDCl_3_): δ 0.92, 1.06 (each d, each 3H, each J = 6.78 Hz, CH(CH_3_)_2_), 1.13 (s, 3-H, 2-CH_3_), 1.43 (s, 12-H, 2-CH_3_, C(CH_3_)_3_), 2.00-2.19 (m, 1H, CH(CH_3_)_2_), 1.86 (br, 1H, NH), 3.37 (d, 1H, J = 4.26 Hz, N-CH-CO), 3.43 (br, 2-H, 3-H, 4-H), 4.17 (br, 1H, OH), 6.74 (d, 1H, J = 8.54 Hz, 8-H), 7.34 (dd, 1H, J = 8.53 Hz, 1.75 Hz, 7-H), 7.69 (d, 1H, J = 1.75 Hz, 5-H); ^13^C NMR (200 MHz, CDCl_3_): δ 17.69 (CH-CH_3_), 18.81 (2-CH_3_), 19.49 (CH-CH_3_), 26.70 (2-CH_3_), 27.92 (C(CH_3_)_3_)_,_ 32.55 (CH(CH_3_)_2_), 56.68 (4-C), 66.54 (N-CH-CO), 74.24 (3-C), 79.72 (C(CH_3_)_3_), 82.13 (2-C), 103.33(6-C), 117.91 (8-C), 119.28 (CN), 126.38 (4a-C), 132.30 (5-C, 7-C), 156.46 (8a-C), 175.98 (C=O); MS m/z 375 (0.18, M+), 273 (28.7%), 160 (32%); Anal. Calcd for C_21_H_30_N_2_O_4_ %: C 67.35, H 8.07, N 7.48; found: C 67.65, H 8.36, N 7.38.

**General procedure for the synthesis of N-methyl derivatives of tert-butyl esters (8a–9b, 11a,b).** The suspension of corresponding amino acid-tert-buty ester (**5a-6b**, **10a,b**) with paraformaldehyde and sodium cyanoborohydride in acidic menthol was stirred and refluxed at room temperature for 4 days, then evaporated in vacuo. Flash chromatography (petroleum ether: ethyl acetate/ 6:1:2).

**(3S,4R)-N-methyl-(6-cyano-3-hydroxy-2,2-dimethyl-2H-3,4-dihydro-1-benzopyrane-4-yl)-L-alanine-tert-butyl-ester (8a).** From **5a,** 81% of **8a** was yielded as yellowish oil; [α]^20^ = (+) 6.92 (c = 0.325, in CH_2_Cl_2_); ^1^H NMR (200 MHz, CDCl_3_)**:** δ 1.21, 1.50 (each s, each 3H, 2x2-CH_3_), 1.52 (s, 9-H, C(CH_3_)_3_), 1.55 (d, 3-H, J = 5.56 Hz, CH-CH_3_), 2.17 (s, 3-H, N-CH_3_), 3.77 (br, 2H, 3-H, 4-H), 3.84 (q, 1H, J = 5.56 Hz, CH-CH_3_), 5.52 (br, 1H, OH), 6.82 (d, 1H, J = 8.46 Hz, 8-H), 7.39 (dd, 1H, J = 2.02 Hz, 8.46 Hz, 7-H), 7.65 (d, 1H, J = 2.02 Hz, 5-H); ^13^C NMR (CDCl_3_):δ 17.11, 18.47 (2x2-CH_3_), 26.76 (CH-CH_3_), 27.63 (C(CH_3_)_3_), 63.42 (4-C), 65.24 (N-CH-CO), 68.76 (3-C), 79.66 (C(CH_3_)_3_), 82.33 (2-C), 103.12 (6-C), 117.90 (8-C), 119.22 (CN), 123.96 (4a-C), 132.04*, 132.52* (5-C, 7-C), 157.54 (8a-C), 175.67 (C=O); MS m/z 360 (0.35, M+), 259 (52.7), 160 (23.5); Anal. Calcd for C_20_H_28_N_2_O_4_ %: C 66.64, H 7.83, N 7.77; found: C 66.38, H 7.69, N 7.73.

**(3R,4S)-N-methyl-(6-cyano-3-hydroxy-2,2-dimethyl-2H-3,4-dihydro-1-benzopyrane-4-yl)-L-alanine-tert-butyl-ester (8b).** From 5b, 78% of 8b was yielded as colorless oil; [α]^20^ = (-) 40.31 (c = 0.129, in CH_2_Cl_2_); ^1^H NMR (200 MHz, CDCl_3_): δ 1.20 (s, 3H, 2-CH_3_), 1.42 (d, 3H, J = 7.45 Hz, CH-CH_3_), 1.45 (s, 9-H, C(CH_3_)_3_), 1.50 (s, 3-H, 2-CH_3_), 2.44 (s, 3-H, N-CH_3_), 3.60 (dd, 1H, J = 2.15 Hz, 9.85 Hz, 3-H), 3.91 (d, 1H , J = 9.85, 4-H), 5.62 (br, 1H, OH), 6.78 (d, 1H, J = 8.59 Hz, 8-H), 7.37 (dd, 1H, J = 2.15 Hz, 8.46 Hz, 7-H), 7.63 (d, 1H, J = 2.15 Hz, 5-H); ^13^C NMR (CDCl_3_): δ 16.69 , 18.86 (2x2-CH_3_), 27.13 (CH-CH_3_), 27.91 (C(CH_3_)_3_), 34.96 (N-CH_3_), 58.66 (4-C), 59.61 (N-CH-CO), 70.05 (3-C), 80.21 (C(CH_3_)_3_), 82.45 (2-C), 103.40 (6-C), 118.01 (8-C), 119.63 (CN), 125.11 (4a-C), 132.25*, 132.83* (5-C, 7-C), 158.08 (8a-C), 177.79 (C=O); MS m/z 361 (0.35, M+1), 259 (53.5%); Anal. Calcd for C_20_H_28_N_2_O_4_ %: C 66.64, H 7.83, N 7.77; found: C 66.76, H 7.55, N 7.65.

**(3S,4R)-N-methyl-(6-cyano-3-hydroxy-2,2-dimethyl-2H-3,4-dihydro-1-benzopyrane-4-yl)-L-valine-tert-butyl-ester (9a).** From 6a, 96% of 9a was yielded as colorless oil; [α]^20^ = (+) 25 (c = 0. 252, in CH_2_Cl_2_); ^1^H NMR (200 MHz, CDCl_3_): δ 0.97, 1.09 (each d, each 3H, each J = 6.70 Hz, CH(CH_3_)_2_), 1.12 (s, 3-H, 2-CH_3_), 1.45 (s, 12H, 2-CH_3_, C(CH_3_)_3_), 2.09 (s, 3H, N-CH_3_), 2.11-2.27 (m, 1H, CH(CH_3_)_2_), 3.34 (d, 1H, J = 7.83 Hz, N-CH-CO), 3.68 (d, 2-H, J = 9.59 Hz, 4-H), 3.74 (dd, 1H, J = 9.54 Hz, 2.40 Hz, 3-H), 4.74 (d, 1H, J = 2.4 Hz, OH), 6.76 (d, 1H, J = 8.46 Hz, 8-H), 7.34 (dd, 1H, J = 8.46 Hz, 2.02 Hz, 7-H), 7.82 (d, 1H, J = 2.02 Hz, 5-H); ^13^C NMR (CDCl_3_): δ 18.49 (CH-CH_3_), 19.49, 19.85 (CH-CH_3_, 2-CH_3_), 26.91 (2-CH_3_), 27.91(C(CH_3_)_3,_ CH(CH_3_)_2_), 29.83 (N-CH_3_), 65.15 (4-C), 68.28 (N-CH-CO), 74.02 (3-C), 79.68 (C(CH_3_)_3_), 82.61 (2-C), 103.44 (6-C), 118.24 (8-C), 119.43 (CN), 124.53 (4a-C), 132.22, 132.84 (5-C, 7C), 157.67 (8a-C), 174.30 (C=O); MS m/z 338 (0.12%, M+), 287 (36%), 160 (17%); Anal. Calcd for C_22_H_32_N_2_O_4_ %: C 68.01, H 8.30, N 7.21; found: C 68.76, H 6.66, N 6.86.

**(3R,4S)-N-methyl-(6-cyano-3-hydroxy-2,2-dimethyl-2H-3,4-dihydro-1-benzopyrane-4-yl)-L-valine-tert-butyl-ester (9b).** From 6b, 84% of 9b was yielded as colorless oil; [α]^20^ = (-) 20.94 (c = 0. 117, in CH_2_Cl_2_); ^1^H NMR (200 MHz, CDCl_3_): δ 0.95, 1.06 (each d, each 3H, each J = 6.57 Hz, CH(CH_3_)_2_), 1.17 (s, 3-H, 2-CH_3_), 1.46 (s, 12-H, 2-CH_3_, C(CH_3_)_3_), 2.28 (s, 3H, N-CH_3_), 2.07-2.22 (m, 1H, CH(CH_3_)_2_), 3.14 (d, 1H, J = 8.97 Hz, N-CH-CO), 3.92 (d, 1H, J = 9.51 Hz, 4-H), 3.61 (dd, 1H, J = 9.51 Hz, 2.27 Hz, 3-H), 5.48 (br, 1H, OH), 6.76 (d, 1H, J = 8.46 Hz, 8-H), 7.34 (dd, 1H, J = 8.46 Hz, 2.02 Hz, 7-H), 7.65(s, 1H, 5-H); ^13^C NMR (CDCl_3_): δ 18.66 (CH-CH_3_), 19.59 (2-CH_3_), 21.12 (CH-CH_3_), 27.05 (2-CH_3_), 27.91 (C(CH_3_)_3_, 28.22 (CH(CH_3_)_2_), 33.89 (N-CH_3_), 58.88 (4-C), 69.20 (N-CH-CO), 72.36 (3-C), 79.94 (C(CH_3_)_3_), 82.64 (2-C), 103.38 (6-C), 118.09 (8-C), 119.46 (CN), 124.53 (4a-C), 132.17, 133.01 (5-C, 7-C), 158.11 (8a-C), 177.34 (C=O); MS m/z 338 (0.10%, M+), 287 (31%), 160 (15.3%); Anal. Calcd for C_22_H_32_N_2_O_4_ %: C 68.01, H 8.30, N 7.21; found: C 68.05, H 8.32, N 6.97.

**(3S,4R)-N-methyl-(6-cyano-3-hydroxy-2,2-dimethyl-2H-3,4-dihydro-1-benzopyrane-4-yl)-D-valine-tert-butyl-ester (11a).** From 10a, 96% of 11a was yielded as colorless oil; [α]^20^ = (+) 19.05 (c = 0. 273, in CH_2_Cl_2_); ^1^H NMR (200 MHz, CDCl_3_): δ 0.96, 1.07 (each d, each 3H, each J = 6.63 Hz, CH(CH_3_)_2_), 1.17 (s, 3H, 2-CH_3_), 1.47 (s, 12H, 2-CH_3_, C(CH_3_)_3_), 2.28 (s, 3H, N-CH_3_), 2.08-2.22 (m, 1H, CH(CH_3_)_2_), 3.16 (d, 1H, J = 8.97 Hz, N-CH-CO), 3.62 (d, 1H, J = 9.54 Hz, 3-H), 3.93 (d, 1H, J = 9.54 Hz, 4-H), 5.54 (br, 1H, OH), 6.77 (d, 1H, J = 8.46 Hz, 8-H), 7.35 (dd, 1H, J = 8.46 Hz, 1.64 Hz, 7-H), 7.66 (s, 1H, 5H); ^13^C NMR (CDCl_3_): δ 18.59 (CH-CH_3_), 19.59 (2-CH_3_), 21.13 (CH-CH_3_), 27.06 (2-CH_3_), 27.94 (C(CH_3_)_3_)_,_ 28.24 (CH(CH_3_)_2_), 33.89 (N-CH_3_), 58.81 (4-C), 69.24 (N-CH-CO), 72.41 (3-C), 79.96 (C(CH_3_)_3_), 82.73 (2-C), 103.30 (6-C), 118.13 (8-C), 119.50 (CN), 124.54 (4a-C), 132.23, 133.04 (5-C, 7-C), 158.16 (8a-C), 177.42 (C=O); MS m/z 338 (0.14%, M+), 287 (56.6%), 160 (22.3%); Anal. Calcd for C_22_H_32_N_2_O_4_ %: C 68.01, H 8.30, N 7.21; found: C 68.24, H 8.32, N 7.47.

**(3R,4S)-N-methyl-(6-cyano-3-hydroxy-2,2-dimethyl-2H-3,4-dihydro-1-benzopyrane-4-yl)-D-valine-tert-butyl-ester (11b).** From 10b, 92% of 11b was yielded as colorless oil; [α]^20^ = (-) 25.79 (c = 0.126, in CH_2_Cl_2_); ^1^H NMR (200 MHz, CDCl_3_): δ 0.98, 1.11 (each d, each 3H, each J = 6.70 Hz, CH(CH_3_)_2_), 1.14 (s, 3H, 2-CH_3_), 1.46 (s, 12H, 2-CH_3_, C(CH_3_)_3_), 2.10 (s, 3H, N-CH_3_), 2.15-2.26 (m, 1H, CH(CH_3_)_2_), 3.36 (d, 1H, J = 7.7 Hz, N-CH-CO), 3.69 (dd, 1H, J = 9.73 Hz, 2.27 Hz, 3-H), 3.75 (d, 1H, J = 9.73 Hz, 4-H), 4.77 (d, 1H, J = 2.27 Hz, OH), 4.77 (d, 1H, J =8.50 Hz, 8-H), 7.35 (dd, 1H, J = 8.50 Hz, 2.00 Hz, 7-H), 7.83 (s, 1H, J = 2.00 Hz, 5H); ^13^C NMR (CDCl_3_): δ 18.54 (CH-CH_3_), 19.54 (2-CH_3_), 19.88 (CH-CH_3_), 26.96 (2-CH_3_), 27.97 (C(CH_3_)_3_)_,_ (CH(CH_3_)_2_), 29.89 (N-CH_3_), 65.24 (4-C), 68.34 (N-CH-CO), 74.08 (3-C), 79.73 (C(CH_3_)_3_), 82.71 (2-C), 103.48 (6-C), 118.29 (8-C), 119.50 (CN), 124.56 (4a-C), 132.28, 132.91 (5-C, 7-C), 157.73 (8a-C), 174.39 (C=O); MS m/z 338 (0.17%, M+), 287 (46.6%), 160 (22%); Anal. Calcd for C_22_H_32_N_2_O_4_ %: C 68.01, H 8.30, N 7.21; found: C 68.24, H 8.55, N 6.99.

**General procedure for cyclization (12a-18b).** 4.61 mmol of amino acid-tert-butyl ester (5a,b-11a,b) was dissolved in a small amount of CH_2_Cl_2_ , hydrolyzed by 6 mL of 70% HClO_4_, stirred overnight, and 4N NH_4_OH solution was added slowly. The precipitate was dried and used in the next reaction step without further purification. A suspension of precipitates (2.76 mmol), 4-dimethylaminopyridine (0.69 mmol) and bis (2-oxo-3oxazolidinyl) phosphinic chloride (4.12 mmol) in CH_2_Cl_2_ (50 mL) was heated to reflux at 80°C for 10 min, then triethylamine (0.95 mL, 6.85 mmol) was added and the solution was refluxed at 70°C for 4 days. The suspension was filtered and evaporated to dryness. Purification was done by flash chromatography (petroleum ether/ethylacetate; 9:1) to yield the target compounds (**12a-18b**).

(2S,4aS,10bR)-N-methyl-2,5,5-trimethyl-3-oxo-1,4a,5,10b-tetrahydro-3H[1] benzopyrano[3,4-b][1,4]oxazine-9-carbonitril (15a). From 8a, 51% of 15a was yielded as colorless crystal; mp 120-121^o^C; [α]^20^ = (+) 112.40 (c = 0.121, in CH_2_Cl_2_); ^1^H NMR (200 MHz, CDCl_3_): δ 1.30 (s, 3H, 5-CH_3_), 1.51 (d, 3H, J = 7.2 Hz, 2-CH_3_), 1.54 (s, 3H, 5-CH_3_), 2.20 (s, 3H, N-CH_3_), 3.95 (q, 1H, J = 7.2 Hz, N-CH-CO), 4.19 (d, 1H, J = 11.24 Hz, 4a-H), 4.51 (d, 1H, J = 11.24 Hz, 10b-H), 6.87 (d, 1H, J = 8.53 Hz, 7-H), 7.45 (dd, 1H, J = 1.87 Hz, 8.53 Hz, 8-H), 7.75 (d, 1H, J = 1.87 Hz, 10-H); ^13^C NMR (CDCl_3_) δ 14.93, 19.66 (2x5-CH_3_), 26.05 (2-CH_3_), 31.11 (N-CH_3_), 56.24 (10b-C), 61.14 (2-C), 74.50 (4a-C), 78.19 (5-C), 104.47 (9-C), 118.51 (7-C), 118.88 (CN), 120.08 (10a-C), 131.53*, 133.19* (8-C, 10-C), 156.61 (6a-C), 171.49 (C=O); MS m/z 286 (1.00, M+), 185 (22%), 170 (100%); Anal. Calcd for C_16_H_18_N_2_O_3_ %: C, 67.12, H 6.34, N 9.78: found: C 67.20, H 6.29, N 9.75.

(2S,4aR,10bS)-N-methyl-2,5,5-trimethyl-3-oxo-1,4a,5,10b-tetrahydro-3H[1] benzopyrano[3,4-b][1,4]oxazine-9-carbonitril (15b). From 8b, 62% of 15b was obtained as yellow crystal; mp 160-165 ^o^C; [α]^20^ = (-) 114.91 (c = 0.108, in CH_2_Cl_2_); ^1^H NMR (200 MHz, CDCl_3_): δ 1.29 (s, 3H, 5-CH_3_), 1.54 (d, 3H, J = 4.8 Hz, 2-CH_3_), 1.56 (s, 3H, 5-CH_3_), 2.32 (s, 3-H, N-CH_3_), 3.60 (q, 1H J = 7.33 Hz, 2-H), 4.11 (d, 1H , J = 10.99 Hz, 4a-H), 4.51 (d, 1H, J = 10.99 Hz, 10b-H), 6.87 (d, 1H, J = 8.56 Hz, 7-H), 7.45 (dd, 1H , J = 1.96 Hz, 8.56 Hz, 8-H), 7.72 (d, 1H, J = 1.96 Hz, 10-H); ^13^C NMR (CDCl_3_): δ 18.69, 19.43 (2x5-CH_3_), 26.05 (2-CH_3_), 37.26 (N-CH_3_), 51.23 (10b-C), 60.49 (N-CH-CO), 73.92 (4a-C), 78.03 (5-C), 104.50 (9-C), 118.46 (7-C), 118.87 (CN), 120.37 (10a-C), 131.66*, 133.11* (8-C, 10-C), 156.69 (6a-C), 171.83 (C=O); MS m/z 286. (1.86, M+), 185 (21%), 170 (100%); Anal.Calcd for C_16_H_18_N_2_O_3_ %: C 67.12, H 6.34, N 9.78; found: C 67.26, H 6.50, N 9.36.

**(2S,4aS,10bR)-2-isopropyl-1,5,5-trimethyl-3-oxo-1,4a,5,10b-tetrahydro-3H[1]benzopyrano[3,4-b][1,4]oxazine-9-carbonitril (16a).** From **9a,** 57% of **16a** was yielded as yellowish oil; [α]^20^ = (+) 119.57 (c = 0.115, in CH_2_Cl_2_); ^1^H NMR (200 MHz, CDCl_3_): δ 0.97, 1.06 (each d, each 3H, J = 6.57 Hz, CH(CH_3_)_2_), 1.38 (s, 3H, 5-CH_3_), 1.54 (s, 3H, 5-CH_3_), 1.63-1.84 (m, 1H, CH-(CH_3_)_2_), 2.71 (s, 3H, N-CH_3_), 2.92 (d, 1H, J = 10.24 Hz, N-CH-CO), 3.61 (d, 1H, J = 11.75 Hz, 4a-H), 4.26 (d, 1H, J = 11.75 Hz, 10b-H), 6.83 (d, 1H, J = 8.56 Hz, 7-H), 7.44 (dd, 1H, J = 8.56 Hz, 2.02 Hz, 8-H), 7.72 (br, 1H, 10H); ^13^C NMR(CDCl_3_): δ 19.30 (CH-CH_3_), 20.40, 21.19 (5-CH_3_, CH-CH_3_), 27.14 (5-CH_3_), 32.55 (CH(CH_3_)_2_), 47.14 (N-CH_3_), 57.05 (10b-C), 73.27 (N-CH-CO), 76.13 (4a-C), 78.58 (5-C), 104.17 (9-C), 117.83 (7-C), 119.23 (CN), 123.90 (10a-C), 130.95, 132.97 (8-C, 10-C), 155.59 (6a-C), 170.31 (C=O); MS m/z 314 (0.41%, M+), 185 (38.5%), 170 (92.4%); Anal. Calcd for C_18_H_22_N_2_O_3_ %: C, 68.77, H 7.05, N 8.91: found: C 68.65, H 7.11, N 8.47.

**(2S,4aR,10bS)-2-isopropyl-1,5,5-trimethyl-3-oxo-1,4a,5,10b-tetrahydro-3H[1]benzopyrano[3,4-b][1,4]oxazine-9-carbonitril (16b).** From **9b,** 52% of **16b** was obtained as yellowish solid; mp 124-125 ^o^C; [α]^20^ = (-) 119.35 (c = 0.124, in CH_2_Cl_2_); ^1^H NMR (200 MHz, CDCl_3_): δ 1.07, 1.14 (each d, each 3H, J = 6.69 Hz, CH(CH_3_)_2_), 1.26 (s, 3H, 5-CH_3_), 1.55 (s, 3H, 5-CH_3_), 2.30 (s, 3H, N-CH_3_), 2.35-2.45 (m, 1H, CH(CH_3_)_2_), 3.17(d, 1H, J = 5.94 Hz, N-CH-CO), 4.07 (d, 1H, J = 10.55 Hz, 4a-H), 4.54 (d, 1H, J = 10.55 Hz, 10b-H), 6.88 (d, 1H, J = 8.58 Hz, 7-H), 7.46 (dd, 1H, J = 8.56 Hz, 1.64 Hz, 8-H), 7.78 (br, 1H, 10-H); ^13^C NMR(CDCl_3_): δ 19.06 (CH(CH_3_)_2_), 19.99 × 25.99 (2×5-CH_3_), 31.81 (CH(CH_3_)_2_), 37.93 (N-CH_3_), 53.21 (10b-C), 70.86 (N-CH-CO), 73.63 (4a-C), 78.01 (5-C), 104.62 (9-C), 118.70 (7-C), 118.95 (CN), 120.26 (10a-C), 131.81, 133.10 (8-C, 10-C), 156.73 (6a-C), 170.73 (C=O); MS m/z 314 (7.3%, M+), 243 (31%), 170 (100%); Anal. Calcd for C_18_H_22_N_2_O_3_ %: C 61.62, H 6.61, N, 7.98; found: C 60.01, H 6.11, N 7.47.

**(2R,4aR,10bS)-2-isopropyl-5,5-dimethyl-3-oxo-1,4a,5,10b-tetrahydro-3H[1]benzopyrano[3,4-b][1,4]oxazine-9-carbonitril (17b).** From **10b,** 45% of **17b** was yielded as pale yellowish crystal; mp 164.5-166^o^C; [α]^20^ = (-) 84.81 (c = 0.158, in CH_2_Cl_2_); ^1^H NMR (200 MHz, CDCl_3_): δ 1.00, 1.12 (each d, each 3H, each J = 7.08 Hz, CH(CH_3_)_2_), 1.29 (s, 3H, 5-CH_3_), 1.52 (s, 3H, 5-CH_3_), 2.47-2.62 (m, 1H, CH(CH_3_)_2_), 3.86 (br, 1H, 10b-H), 3.96 (d, 1H, J = 5.94 Hz, N-CH-CO), 4.06 (d, 1H, J = 9.98 Hz, 4a-H), 6.87 (d, 1H, J = 8.59 Hz, 7-H), 7.46 (dd, 1H, J = 8.59 Hz, 2.02 Hz, 8-H), 7.82 (d, 1H, J = 2.02 Hz, 10-H); ^13^C NMR (CDCl_3_): δ 17.42 (CH-CH_3_), 19.01 (5-CH_3_), 25.87 (5-CH_3_), 19.80 (CH-CH_3_), 30.98 (CH(CH_3_)_2_), 49.28 (10b-C), 63.73 (N-CH-CO), 77.95 (5-C), 83.03 (4a-C), 104.23 (9-C), 118.25 (7-C), 118.88 (CN), 121.34 (10a-C), 131.30, 133.37 (8-C, 10-C), 155.96 (6a-C), 169.47 (C=O); MS m/z 300 (0.57%, M+), 185 (38.5%), 170 (100%); Anal. Calcd for C_17_H_20_N_2_O_3_ %: C 67.98, H 6.71, N 9.33; found: C 68.28, H 6.91, N 9.04.

**(2R,4aS,10bR)-2-isopropyl-1,5,5-trimethyl-3-oxo-1,4a,5,10b-tetrahydro-3H[1]benzopyrano[3,4-b][1,4]oxazine-9-carbonitril (18a).** From **11a,** 45% of **18a** was obtained as yellowish solid; mp: 151-153 °C; [α]^20^ = (+) 107.17 (c = 0.30, in CH_2_Cl_2_); ^1^H NMR (200 MHz, CDCl_3_): δ 1.07, 1.14 (each d, each 3H, J = 6.69 Hz, CH(CH_3_)_2_), 1.26 (s, 3H, 5-CH_3_), 1.56 (s, 3H, 5-CH_3_), 2.30 (s, 3-H, N-CH_3_), 2.35-2.45 (m, 1H, CH(CH_3_)_2_), 3.17 (d, 1H, J = 5.93 Hz, N-CH-CO), 4.07 (d, 1H, J = 10.67 Hz, 4a-H), 4.54 (d, 1H, J = 10.67 Hz, 10b-H), 6.89 (d, 1H, J = 8.53 Hz, 7-H), 7.46 (dd, 1H, J = 8.53 Hz, 1.51 Hz, 8-H); 7.78 (br, 1H, 10-H); ^13^C NMR (CDCl_3_): δ 19.08 (CH(CH_3_)_2_), 20.00 (5-CH_3_), 25.99 (5-CH_3_), 31.82 (CH(CH_3_)_2_), 37.94 (N-CH_3_), 53.19 (10b-C), 70.86 (N-CH-CO), 73.64 (4a-C), 78.01 (5-C), 104.63 (9-C), 118.71 (7-C), 118.95 (CN), 120.27 (10a-C), 131.82, 133.11 (8-C, 10-C), 156.74 (6a-C), 170.72 (C=O); MS m/z 314 (5.36%, M+), 243 (18.2%), 170 (100%); Anal. Calcd for C_18_H_22_N_2_O_3_ %: C 68.77, H 7.05, N 8.91; found: C 68.32, H 7.06, N 8.71.

**(2R,4aR,10bS)-2-isopropyl-1,5,5-trimethyl-3-oxo-1,4a,5,10b-tetrahydro-3H[1]benzopyrano[3,4-b][1,4]oxazine-9-carbonitril (18b).** From **11b,** 52% of **18b** was yielded as yellowish oil; [α]^20^ = (-) 125.71 (c = 0.105, in CH_2_Cl_2_); ^1^H NMR (200 MHz, CDCl_3_): δ 0.97, 1.06 (each d, each 3H, J = 6.57 Hz, CH(CH_3_)_2_), 1.37 (s, 3H, 5-CH_3_), 1.54 (s, 3H, 5-CH_3_), 1.62-1.81 (m, 1H, CH(CH_3_)_2_), 2.71 (s, 3H, N-CH_3_), 2.91 (d, 1H, J = 10.23 Hz, N-CH-CO), 3.60 (d, 1H, J = 11.74 Hz, 4a-H), 4.25 (d, 1H, J = 11.74 Hz, 10b-H), 6.83 (d, 1H, J = 8.56 Hz, 7-H), 7.44 (dd, 1H, J = 8.56 Hz, 1.52 Hz, 8-H); 7.71 (br, 1H, 10-H); ^13^C NMR (CDCl_3_): δ 19.28 (CH-CH_3_), 20.38 (5-CH_3_), 21.17 (CH-CH_3_), 27.12 (5-CH_3_), 32.52 CH(CH_3_)_2_, 47.12 (N-CH_3_), 57.01 (10b-C), 73.27 (N-CH-CO), 76.09 (4a-C), 78.55 (5-C), 104.12 (9-C), 117.80 (7-C), 119.21 (CN), 123.87 (10a- C), 130.93, 132.94 (8-C, 10-C), 155.56 (6a-C), 170.30 (C=O); MS m/z 314 (1.14%, M+), 243 (16.2%), 185 (45%), 170 (100%); Anal. Calcd for C_18_H_22_N_2_O_3_ %: C 68.77, H 7.05, N 8.91; found: C 68.78, H 7.12, N 8.61.

**General procedure for reaction of epoxide with valinol (19a,b).** A solution of enantiomerically pure epoxide (**4a**, **4b**) (4.97mmol) and L-valinol (4.47 mmol) in 50 mL of 96% ethanol was heated to reflux at 65 °C for 5 days. The solvent was removed under reduced pressure. Purification was done by flash column chromatography (petroleum ether: ethylacetate /8:2)**.**

**(2S,3S,4R)-3-hydroxy-4-(1-hydroxy-3-methyl-2-butyl-amino)-2,2-dimethyl-2H-3,4-dihydro-1-benzopyran-6-carbonitril (19a).** Yielded 67% pale yellowish crystal; mp: 141-142 °C; [α]^20^ = (+) 13.45 (c = 0.119, in CH_2_Cl_2_); ^1^H NMR (200 MHz, CDCl_3_): δ 1.00, 1.03 (each d, each 3H, each J = 1.63 Hz, CH(CH_3_)_2_), 1.22 (s, 3H, 2-CH_3_), 1.47 (s, 3H, 2-CH_3_), 1.61 (br, 1H, NH), 1.74-1.91 (m, 1H, CH(CH_3_)_2_), 3.02-3.10 (m, 1H, N-CH), 3.48 (d, 1H, J = 10.04 Hz, 3-H), 3.61 (m, 2H, CH_2_O), 3.86 (dd, 1H, J = 10.04 Hz, 3.26 Hz, 4-H), 4.83 (br, 1H, OH), 6.78 (d, 1H, J = 8.53 Hz, 8-H); 7.38 (dd, 1H, J = 8.53 Hz, 1.51 Hz, 7-H), 7.92 (d, 1H, J = 1.51 Hz, 5-H); ^13^C NMR(CDCl_3_): δ 18.79, 18.98, 19.34 (2-CH_3_, CH(CH_3_)_2_), 26.85 (2-CH_3_), 32.00 (CH(CH_3_)_2_), 57.32 (CH_2_OH), 63.94 (4-C), 64.31 (N-CH), 77.21 (3-C), 97.80 (2-C), 103.30 (6-C), 117.94 (8-C), 119.61 (CN), 127.08 (4a- C), 123.38, 132.69 (5-C, 7-C), 156.65 (8a-C); MS m/z 305 (0.72%, M+), 273 (39.8%), 160 (60.6%); Anal. Calcd for C_17_H_24_N_2_O_3_ %: C 67.08, H 7.95, N 9.20; found: C 67.07, H 7.77, N 9.20.

**(2S,3R,4S)-3-hydroxy-4-(1-hydroxy-3-methyl-2-butyl-amino)-2,2-dimethyl-2H-3,4-dihydro-1-benzopyran-6-carbonitril (19b).** Yielded 65% pale yellowish oil; [α]^20^ = (-) 8.65 (c = 0.104, in CH_2_Cl_2_); ^1^H NMR (200 MHz, CDCl_3_): δ 0.90, 0.94 (each d, each 3H, J = 3.91 Hz, CH(CH_3_)_2_), 1.21 (s, 3H, 2-CH_3_), 1.47 (s, 3H, 2-CH_3_), 1.70-1.86 (m, 1H, CH(CH_3_)_2_), 2.92-3.00 (m, 1H, N-CH), 3.50 (m, 2H, J = 8.65 Hz, CH_2_O), 3.69 (d, 1H, J = 10.05 Hz, 3H), 3.81 (dd, 1H, J = 10.05 Hz, 3.16 Hz, 4H), 6.77 (d, 1H, J = 8.56 Hz, 8-H), 7.36 (dd, 1H, J = 8.56 Hz, 1.84 Hz, 7-H), 8.02 (s, 1H, 5H); ^13^C NMR(CDCl_3_): δ 16.96 (CH-CH_3_), 19.30, 19.58 (2-CH_3,_ CH-CH_3_), 26.63 (2-CH_3_), 30.28 (CH(CH_3_)_2_), 54.71 (4-C), 61.75 (CH_2_OH), 62.24 (N-CH), 73.99 (3-C), 79.58 (2-C) 102.88 (6-C), 117.85 (8-C), 119.48 (CN), 126.41 (4a-C), 132.20, 132.83 (5-C, 7C), 156.81 (8a-C); MS m/z 305 (0.47%, M+), 273 (16.6%), 160 (21.9%); Anal. Calcd for C_17_H_24_N_2_O_3_ %: C 67.08, H 7.95, N 9.20; found: C 67.12, H 7.88, N 9.10.

**General procedure for N-methylation of 3-hydroxy-4-(1-hydroxy-3-methyl-2-butyl-amino)-2,2-dimethyl-2H-3,4-dihydro-1-benzopyran-6-carbonitril.** The suspension of educt**19a,b**, 250 mg of paraformaldehyde and 95% sodium cyanoborohydride (NaBH_3_CN) (326mg) in 30 mL of MeOH was stirred at room temperature overnight. The pH of suspension was adjusted to 6 with glacial acetic acid. Flash chromatography (petroleum ether: ethylacetate /9:1) were used to purify.

**(2S,3S,4R)-3-hydroxy-4-(1-hydroxy-3-methyl-2-butyl-N-methyl-amino)-2,2-dimethyl-2H-3,4-dihydro-1-benzopyran-6-carbonitril (20a).** From **19a** yielded 87% colorless crystal of **20a**; mp 143-144 °C; [α]^20^ = (+) 39.45 (c = 0.109, in CH_2_Cl_2_); ^1^H NMR (200 MHz, CDCl_3_): δ 1.00, 1.03 (each d, each 3H, J = 6.00 Hz, CH(CH_3_)_2_), 1.20 (s, 3H, 2-CH_3_), 1.48 (s, 3H, 2-CH_3_), 1.73-1.89 (m, 1H, CH(CH_3_)_2_), 2.22 (s, 3H, N-CH_3_), 3.07-3.17 (dt, 1H, J = 10.7 Hz, 4.30 Hz, N-CH), 3.74 (d, 1H, J = 9.85 Hz, 4-H), 3.78 (d, 1H, J = 9.85 Hz, 3-H), 3.85 (dd, 1H, J = 10.7 Hz, 4.30 Hz, OCH_A_), 3.98 (t, 1H, J = 10.7 Hz, OCH_B_ ), 6.79 (d, 1H, J = 8.46 Hz, 8-H), 7.38 (dd, 1H, J = 8.46 Hz, 1.65 Hz, 7-H), 7.96 (d, 1H, J = 1.65 Hz, 5H); ^13^C NMR (CDCl_3_): δ 19.03 (CH-CH_3_), 19.95, 20.07 (2-CH_3,_ CH-CH_3_), 27.17 (2-CH_3_), 28.64 (N-CH_3_), 32.98 (CH(CH_3_)_2_), 62.43 (CH_2_OH), 66.04 (4-C), 69.08 (3-C), 69.23 (N-CH), 79.63 (C(CH_3_)_3_), 103.28 (6-C), 118.17 (8-C), 119.88 (CN), 125.12 (4a-C), 132.23, 133.01 (5-C, 7-C), 158.01 (8a-C); MS m/z 318 (0.38%, M+), 160 (43.2%), 86 (100%); Anal. Calcd for C_18_H_26_N_2_O_3_ %: C 67.90, H 8.23, N 8.80; found: C 68.04, H 8.01, N 8.97.

**(2S,3R,4S)-3-hydroxy-4-(1-hydroxy-3-methyl-2-butyl-N-methyl-amino)-2,2-dimethyl-2H-3,4-dihydro-1-benzopyran-6-carbonitril (20b).** From **19b** yielded 95% pale yellowish solid of **20b**; mp: 97-100 °C; [α]^20^ = (-) 20.91 (c = 0.112, in CH_2_Cl_2_); ^1^H NMR (200 MHz, CDCl_3_): δ 0.92, 1.02 (each d, each 3H, J = 6.82 Hz, CH(CH_3_)_2_), 1.20 (s, 3H, 2-CH_3_), 1.48 (s, 3H, 2-CH_3_), 2.00-2.16 (m, 1H, CH(CH_3_)_2_), 2.32 (s, 3H, N-CH_3_), 2.80-2.88 (m, 1H, N-CH), 3.72 (d, 1H, J = 10.1 Hz, 4-H), 3.78 (d, 1H, J = 11.56 Hz, OCH_A_), 3.92 (dd, 1H, J = 11.56 Hz, 2.21 Hz, OCH_B_ ), 4.08 (d, 1H, J = 10.01 Hz, 3H), 6.78 (d, 1H, J = 8.46 Hz, 8H), 7.37 (dd, 1H, J = 8.46 Hz, 1.90 Hz, 7-H), 7.90 (d, 1H, J = 1.90 Hz, 5-H); ^13^C NMR (CDCl_3_): δ 17.43 (CH-CH_3_), 19.07 (2-CH_3_), 21.22 (CH-CH_3_), 27.08 (2-CH_3_), 28.97 (N-CH_3_), 34.90 (CH(CH_3_)_2_), 54.41 (4-C), 60.64 (CH_2_OH), 69.81 (3-C), 70.65 (N-CH), 80.21 (2-C), 103.07 (6-C), 117.93 (8-C), 119.83 (CN), 125.65 (4a-C), 132.20, 133.12 (5-C, 7-C), 157.99 (8a-C); MS m/z 318 (0.35%, M+), 160 (43.5%), 86 (100%); Anal. Calcd for C_18_H_26_N_2_O_3_ %: C 67.90, H 8.23, N 8.80; found: C 67.93, H 8.14, N 8.73.

**General procedure for ring closure via intramolecular ether formation (21a,b).** A suspension of **19a,b** (600mg), trimethylamine (0.44mL) and 0.2 mg of trimethylamine hydrochloride in 2 mL of toluene was cooled at 0 °C in an ice-acetone bath, than added cooled solution of methane sulfonyl chloride (4.36 mmol) in toluene and stirred at 0°C for 1.5 hr. The solvent was removed under reduced pressure. The residue was dissolved in 10 mL of tetrahydrofuran, and 60% sodium hydride dispersion in mineral oil (6.25 mmol) was added bit by bit, and then stirred overnight. The residue was purified by flash column chromatography (petroleum ether: ethylacetate /9:1). Surprisingly, in case of the tertiary amine (**20a,b**), the cyclisation failed and the respective chloro-derivatives **22a,b** were obtained.

**(2S,4aS,10bR)-2-isopropyl-5,5-dimethyl-1,4a,5,10b-tetrahydro-3H [1]benzopyrano[3,4-b][1,4]oxazine-9-carbonitril (21a).** Yielded 50% yellowish oil; ^1^H NMR (200 MHz, CDCl_3_): δ 0.98, 1.02 (each d, each 3H, each J = 2.66 Hz, CH(CH_3_)_2_), 1.25 (s, 3H, 5-CH_3_), 1.66 (s, 3H, 5-CH_3_), 1.76- 1.83 (m, 1H, 2H), 1.99-2.15 (m, 1H, CH(CH_3_)_2_), 2.50 (d, 1H, J = 6.83 Hz, 4a-H), 2.56 (d, 1H, J = 6.83 Hz, 10b-H), 3.69 (d, 2-H, J = 4.68 Hz, 3-CH_2_), 6.86 (d, 1H, J = 8.37 Hz, 7-H), 7.47 (dd, 1H, J = 8.37 Hz, 2.02 Hz, 8-H), 7.57 (d, 1H, J = 2.02 Hz, 10-H); ^13^C NMR (CDCl_3_): δ 19.19, 19.27 (CH(CH_3_)_2_), 24.47 (5-CH_3_), 26.12 (5-CH_3_), 31.32 (CH(CH_3_)_2_), 37.45 (10b-C), 45.98 (3-C), 48.90 (2-C), 73.95 (5-C), 74.26 (4a-C), 103.85 (9-C), 119.21(7-C), 123.98 (10a-C), 132.51, 132.70 (8-C, 10-C), 156.12 (6a-C); MS m/z 269 (54.4%, M+), 170 (22.9%), 157 (75.4%); Anal. Calcd for C_17_H_22_N_2_O_2_ %: C 71.30, H 7.78, N 9.78; found: C 71.12, H 7.65, N 9.85.

**(2S,4aR,10bS)-2-isopropyl-5,5-dimethyl-1,4a,5,10b-tetrahydro-3H [1]benzopyrano[3,4-b][1,4]oxazine-9-carbonitril (21b).** Yielded 51% yellowish oil; ^1^H NMR (200 MHz, CDCl_3_): δ = 0.98, 1.10 (each d, each 3H, J = 6.26 Hz, CH(CH_3_)_2_), 1.25 (s, 3H, 5-CH_3_), 1.44 (s, 3H, 5-CH_3_), 2.15-2.29 (m, 1H, CH(CH_3_)_2_), 2.36 (dd, 1H, J = 10.36 Hz, 2.91 Hz, 2-H), 3.08 (d, 1H, J = 10.11 Hz, 4a-H), 3.62 (dd, 1H, J = 11.75 Hz, 3.03 Hz, 3-CH_2A_), 3.80 (d, 1H, J = 10.11 Hz, 10b-H), 4.16 (d, 1H, J = 11.87 Hz, 3-CH_2B_), 6.79 (d, 1H, J = 8.59 Hz, 7-H), 7.39 (dd, 1H, J = 8.59 Hz, 1.51 Hz, 8-H), 7.69 (d, 1H, J = 1.51 Hz, 10-H); ^13^C NMR(CDCl_3_): δ 19.86 (CH-CH_3_), 20.70, 20.81 (CH-CH_3,_ 5-CH_3_), 25.80 (CH(CH_3_)_2_), 26.69 (5-CH_3_), 45.36 (10b-C), 58.77 (2-C), 69.40 (3-C), 78.79 (5-C), 81.88 (4a-C), 103.41 (9-C), 117.75 (7-C), 119.35 (CN), 124.10 (10a-C), 130.06, 132.51 (8-C, 10-C), 156.44 (6a-C); MS m/z 286 (11.9%, M+), 243 (100%), 170 (25%); Anal. Calcd for C_17_H_22_N_2_O_2_ %: C 71.30, H 7.74, N 9.78; found: C 71.45, H 7.64, N 9.61.

**(2S,3S,4R)-4-(N-(1-chloro-3-methyl-2-butyl-N-methyl-amino)-3-hydroxy-2,2-dimethyl-2H-3,4-dihydro-1-benzopyran-6-carbonitril (22a).** From **20a** yielded 55% yellowish oil of **22a**; [α]^20^ = (-) 7.77 (c = 0.103, in CH_2_Cl_2_); ^1^H NMR ( 200 MHz, CDCl_3_): δ 0.90, 1.02 (each d, each 3H, each J = 6.63 Hz, CH(CH_3_)_2_), 1.18 (s, 3-H, 2-CH_3_), 1.48 (s, 3H, 2-CH_3_), 1.90-2.05 (m, 1H, CH(CH_3_)_2_), 2.51 (s, 3-H, N-CH_3_), 2.95 (br, 2H, CH_2_Cl), 3.24 (br, 1H, OH), 3.61 (d, 1H, J = 10.17 Hz, 3-H), 3.69 (d, 1H, J = 10.17 Hz, 4-H), 3.97-4.05 (m, 1H, N-CH), 6.80 (d, 1H, J = 8.50 Hz, 8-H), 7.36 (dd, 1H, J = 8.50 Hz, 1.77 Hz, 7-H ), 7.64 (br, 1H, 5-H); ^13^C NMR (CDCl_3_): δ 16.64 (2-CH_3_), 18.73 (CH-CH_3_), 20.35 (2-CH_3_), 26.84 (CH-CH_3_), 31.84 (CH(CH_3_)_2_), 38.84 (N-CH_3_ at 60 °C 300 MHz), 60.18 (CH_2_Cl at 60 °C 300 MHz), 63.50 (4-C), 68.54 (N-CH), 70.01 (3C), 79.61 (C(CH_3_)_3_), 103.28 (6-C), 118.48 (8-C), 119.38 (CN), 123.46 (4a-C), 132.39, 132.44 (5-C, 7-C), 157.57 (8a-C); MS m/z 336 (0.43%, M+), 229 (56.5%), 160 (35.1%); Anal. Calcd for C_18_H_25_ClN_2_O_2_ %: C 64.18, H 7.48, N 8.32, Cl 10.52; found: C 63.61, H 7.17, N 7.79, Cl 9.93.

**(2S,3R,4S)-4-(N-(1-chloro-3-methyl-2-butyl-N-methyl-amino)-3-hydroxy-2,2-dimethyl-2H-3,4-dihydro-1-benzopyran-6-carbonitril (22b).** From **20b** yielded 60% yellowish oil of **22b**; [α]^20^ = (-) 31.98 (c = 0.111, in CH_2_Cl_2_); ^1^H NMR ( 200 MHz, CDCl_3_): δ 1.00, 1.08 (each d, each 3H, each J = 6.70 Hz, CH(CH_3_)_2_), 1.19 (s, 3-H, 2-CH_3_), 1.50 (s, 3-H, 2-CH_3_), 1.97-2.12 (m, 1H, CH(CH_3_)_2_), 2.22 (s, 3H, N-CH_3_), 3.12 (dd, 1H, J = 13.64 Hz, 10.23 Hz, CH_2A_-Cl), 3.40 (dd, 1H, J = 14.08 Hz, 7.14 Hz, CH_2B_-Cl), 3.59 (d, 1H, J = 10.04 Hz, 3-H), 3.71 (d, 1H, J = 10.04 Hz, 4-H), 4.02-4.11 (m, 1H, N-CH), 6.84 (d, 1H, J = 8.08 Hz, 8-H), 7.37 (s, 1H, 5-H), 7.39 (d, 1H, J = 8.08 Hz, 7-H); ^13^C NMR (CDCl_3_): δ 16.80 (CH-CH_3_), 18.66 (2-CH_3_), 20.30 (CH-CH_3_), 26.88 (2-CH_3_), 32.07 (CH(CH_3_)_2_), 34.96 (N-CH_3_ at 60 °C 300 MHz), 64.07 (CH_2_Cl at 60 °C 300 MHz), 65.37 (4-C), 69.67 (N-CH), 70.89 (3-C), 79.74 (C(CH_3_)_3_), 103.17 (6-C), 118.83 (8-C), 119.41 (CN), 122.48 (4a-C), 132.20, 132.59 (5-C, 7-C), 157.77 (8a-C); MS m/z 336 (0.51%, M+), 229 (37.08%), 160 (71.2%); Anal. Calcd for C_18_H_25_ClN_2_O_2_ %: C 64.18, H 7.48, N 8.32, Cl 10.52; found: C 64.25, H 7.57, N 7.80, Cl 9.01.

Biological Assay

The human T-lymphoblast cell line CCRF-CEM and the multidrug resistant CEM/vcr1000 cell line were provided by V. Gekeler (Byk Gulden, Konstanz, Germany). The resistant CEM/vcr1000 line was obtained by stepwise selection in vincristine containing medium. Cells were kept under standard culture conditions (RPMI1640 medium supplemented with 10% fetal calf serum). P-gp-expressing resistant cell line was cultured in presence of 1000 ng/mL vincristine. One week prior to the experiments cells were transferred into medium without selective agents or antibiotics. Briefly, cells were pelleted, the supernatant was removed by aspiration and cells were resuspended at a density of 1 x 106/ mL in PRMI1640 medium containing 3µmol/l daunomycin. Cell suspensions were incubated at 37°C for 30 min. After this time a steady state of daunorubicin accumulation was reached. Tubes were chilled on ice and cells were pelleted at 500 x g. Cells were washed once in RPMI1640 medium to remove extracellular daunorubicin. Subsequently, cells were resuspended in medium prewarmed to 37°C, containing either no modulator or chemosensitizer at various concentrations ranging from 3nM to 500 µM, depending on solubility and expected potency of the modifier. Generally, 8 serial dilutions were tested for each modulator. After 1, 2, 3 and 4 min aliquots of the incubation mixture were drawn and pipetted into 4 volumes of ice cold stop solution (RPMI1640 medium containing verapamil at a final concentration of 100 µM). Parental CCRF-CEM cells were used to correct for simple membrane diffusion, which was less than 3% of the efflux rates observed in resistant cells. Samples drawn at the respective time points were kept in an ice water bath and measured within one hour on a Becton Dickinson FACS Calibur (Becton Dickinson, Heidelberg, Germany) flow cytometer as described. Dose response curves were fitted to the data points using non-linear least squares and IC_50_ values were calculated as described by Chiba *et al* [4]. IC_50_ values of individual compounds the average of at least triplicate determinations (Table 1). A CV of below 20% was obtained in all determinations.

SM Table 1 Summary of the 2D-QSAR models developed by leave one pair out cross validation of the enantiomerically pure Benzopyrans and Benzopyrano[3,4b][1,4]-oxazines (5a-22b).

| **Leave one pair out** | **q^2^** | **R^2^** | **RMSE** | **Equation** |
| --- | --- | --- | --- | --- |
| 5a, b out  6a, b out  7a, b out  8a, b out  9a, b out  10 a, b out  11 a, b out  12a, b out  13a, b out  14a, b out  15a, b out  16 a, b out  17b  18a, b out  19a, b out  20a,b out  21 a,b out  22 a,b out | 0.63  0.61  0.57  0.62  0.58  0.58  0.57  0.58  0.64  0.69  0.62  0.63  0.67  0.63  0.63  0.63  0.61  0.70 | 0.67  0.66  0.62  0.66  0.63  0.63  0.63  0.62  0.68  0.73  0.67  0.67  0.71  0.68  0.67  0.67  0.66  0.74 | 0.49  0.50  0.50  0.50  0.50  0.50  0.50  0.47  0.48  0.43  0.48  0.49  0.46  0.49  0.48  0.49  0.50  0.44 | 0.01(vsa_hyd) -4.74  0.01(vsa_hyd) -4.71  0.01(vsa_hyd) -4.60  0.01(vsa_hyd) -4.75  0.01(vsa_hyd) -4.70  0.01(vsa_hyd) -4.68  0.01(vsa_hyd) -4.68  0.01(vsa_hyd) -4.59  0.01(vsa_hyd) -4.94  0.01(vsa_hyd) -4.73  0.01(vsa_hyd) -4.75  0.01(vsa_hyd) -4.73  0.01(vsa_hyd) -4.95  0.01(vsa_hyd) -4.68  0.01(vsa_hyd) -4.74  0.01(vsa_hyd) -4.76  0.01(vsa_hyd) -4.71  0.01(vsa_hyd) -4.88 |

**SM Table 2** Summary of GRIND models developed by leave one pair out cross validation of the enantiomerically pure Benzopyranes and Benzopyrano[3,4b][1,4]-oxazines (**5a-22b**).

| **Leave one pair out** | **q^2^** | **R^2^** | **SDEP** | **Prediction**  **Code Exp. LogIC_50_ Pred. LogIC_50_** | | |
| --- | --- | --- | --- | --- | --- | --- |
| 5a, b out  6a, b out  7a, b out  8a, b out  9a, b out  10 a, b out  11 a, b out  12a, b out  13a, b out  14a, b out  15a, b out  16 a, b out  17b, 18a, b out  19a, b out  20a,b out  21 a,b out  22 a,b out | 0.59  0.59  0.67  0.58  0.59  0.59  0.56  0.58  0.50  0.62  0.56  0.58  0.65  0.57  0.56  0.65  0.62 | 0.81  0.73  0.86  0.72  0.73  0.74  0.71  0.74  0.78  0.82  0.72  0.72  0.87  0.70  0.70  0.85  0.85 | 0.52  0.56  0.43  0.52  0.50  0.51  0.51  0.47  0.57  0.48  0.53  0.52  0.53  0.52  0.54  0.47  0.50 | 5a  5b  6a  6b  7a  7b  8a  8b  9a  9b  10a  10b  11a  11b  12a  12b  13a  13b  14a  14b  15a  15b  16a  16b  17b  18a  18b  19a  19b  20a  20b  21a  21b  22a  22b | -1.47  -1.16  -0.38  -0.43  0.26  0.11  -0.60  -0.57  0.02  -0.13  -0.66  -0.13  -0.004  0.005  -3.09  -1.88  -1.18  -1.77  -0.42  -2.41  -1.68  -1.46  -1.68  -1.22  -0.98  -1.89  -1.44  -1.73  -2.01  -0.74  -0.83  -1.69  -1.64  -1.55  -1.65 | -0.43  -0.18  -0.74  -0.13  -0.60  -0.56  -0.60  -0.89  -0.83  -0.25  -0.51  -0.48  -0.73  -0.66  -1.43  -1.46  -1.79  -2.12  -1.27  -0.81  -1.43  -1.57  -1.54  -1.87  -1.80  -1.60  -1.63  -1.63  -1.25  -0.64  -0.91  -1.50  -1.71  -1.04  -1.24 |

**References**

1. Lee NH, Muci AR, Jacobsen EN (1991) Enantiomerically Pure Epoxychromans via Asymmetric Catalysis. Tetrahedron Letters 32:5055-5058

2. Callahan. FM, Anderson. GW, Paul. R, Zimmerman. J (1963) The Tertiary Butyl Group as a Blocking Agent for Hydroxyl, Sulfhydryl and Amido Functions in Peptide Synthesis. J Am Chem Soc 85:201-207

3. Jabeen I, Wetwitayaklung P, Klepsch F, Parveen Z, Chiba P, Ecker GF (2011) Probing the stereoselectivity of P-glycoprotein-synthesis, biological activity and ligand docking studies of a set of enantiopure benzopyrano[3,4-b][1,4]oxazines. Chem Commun (Camb) 47:2586-2588

4. Chiba P, Ecker G, Schmid D, Drach J, Tell B, Goldenberg S, Gekeler V (1996) Structural requirements for activity of propafenone-type modulators in P-glycoprotein-mediated multidrug resistance. Mol Pharmacol 49:1122-1130
